# Enriching Beneficial Microbial Diversity of Indoor Plants and Their Surrounding Built Environment With Biostimulants

**DOI:** 10.3389/fmicb.2018.02985

**Published:** 2018-12-05

**Authors:** Alexander Mahnert, Marika Haratani, Maria Schmuck, Gabriele Berg

**Affiliations:** Institute of Environmental Biotechnology, Graz University of Technology, Graz, Austria

**Keywords:** indoor plants, built environment, biostimulants, vermicompost, microbiome, 16S rRNA gene amplicon analysis, qPCR, LC-MS

## Abstract

Microbial diversity is suggested as the key for plant and human health. However, how microbial diversity can be enriched is largely unknown but of great interest for health issues. Biostimulants offer the way to directly augment our main living areas by the healthy microbiome of indoor plants. Here, we investigated shifts of the microbiome on leaves of spider plants (*Chlorophytum comosum*) and its surrounding abiotic surfaces in the built environment after irrigation with a vermicompost-based biostimulant for 12 weeks. The biostimulant could not only promote plant growth, but changed the composition of the microbiome and abundance of intact microbial cells on plant leaves and even stronger on abiotic surfaces in close vicinity under constant conditions of the microclimate. Biostimulant treatments stabilized microbial diversity and resulted in an increase of *Bacteroidetes* and a surprising transient emerge of new phyla, e.g., *Verrucomicrobia, Acidobacteria*, and *Thaumarchaeota.* The proportion of potentially beneficial microorganisms like *Brevibacillus, Actinoallomurus, Paenibacillus, Sphaerisporangium* increased relatively; microbial diversity was stabilized, and the built environment became more plant-like. Detected metabolites like indole-3-acetic acid in the biostimulant were potentially contributed by species of *Pseudomonas*. Overall, effects of the biostimulant on the composition of the microbiome could be predicted with an accuracy of 87%. This study shows the potential of biostimulants not only for the plant itself, but also for other living holobionts like humans in the surrounding environment.

## Introduction

Plants are apart from humans and animals often part of indoor environments and provide a sustainable but underexploited solution to enhance indoor air quality (Brilli et al., 2018), and serve as an important source for microbial communities (Berg et al., 2014; Mahnert et al., 2015). Plants themselves possess a unique microbiome, and their functional interplay determines health and productivity of the plant holobiont (Bulgarelli et al., 2013; Vandenkoornhuyse et al., 2015). The plant microbiome varies between different locations on the plant (Turner et al., 2013). For example, the rhizosphere is rich in nutrients derived from root exudates, and represents a relatively stable and protected interface to the surrounding soil (Philippot et al., 2013). The phyllosphere, which represents the air-plant interface, is nutrient-poor and its environment is more dynamic and affected by abiotic factors from the surrounding outdoor environment (Turner et al., 2013). Nevertheless, both microenvironments (rhizosphere and phyllosphere) and their inhabiting microorganisms are connected by the endosphere (Berg et al., 2005; Hardoim et al., 2015). This was also shown in the study of (Badri et al., 2013), where application of soil microbes to the roots resulted in a direct increase in metabolism of the corresponding plant leaves. This phenomenon and the observations that plants can alter microbial abundance and diversity within the built environment (Mahnert et al., 2015) suggest that it might be possible to not only influence the microbiome of a plant, but also their environment by increasing and stabilizing the existing microbial community with them. While the impact of them on the rhizosphere microbiome is well studied (Erlacher et al., 2014; Kröber et al., 2014), less is known about their impact on the phyllosphere and on the environmental microbiome.

Based on the beneficial plant-microbe interactions and mode of action, beneficial microorganisms can be used as biofertilizers, plant strengtheners, biostimulants, and biopesticides (Berg, 2009). Biostimulants have broad applications ranging from enhancing nutrition efficiency, over crop quality till abiotic stress tolerance as reviewed by du Jardin (2015). They can include diverse formulations of compounds, substances and microorganisms that are applied to plants or soils to improve plants vigor, yields, quality and tolerance of abiotic stresses. The mode of action of biostimulants has been associated to direct effects by stimulation of enzyme activities and hormonal activities and also indirectly by improvement of soil nutrient availability. Moreover, a modification of natural microbial communities is suggested, but was never shown. However, the detailed molecular, cellular and physiological mechanisms underlying plant-biostimulant interactions under different environment and management strategies remain largely unknown. Although, the concept of biostimulants based on the principle that biological function can be positively modulated through application of molecules, or mixtures of molecules (Yakhin et al., 2017), an understanding of the mechanism is important for consistent effects. Our hypothesis was that biostimulants have a positive impact on plant growth and performance due to their ability to stabilize the whole plant microbiome, and beyond that of the surrounding microbiome.

**FIGURE 1:**
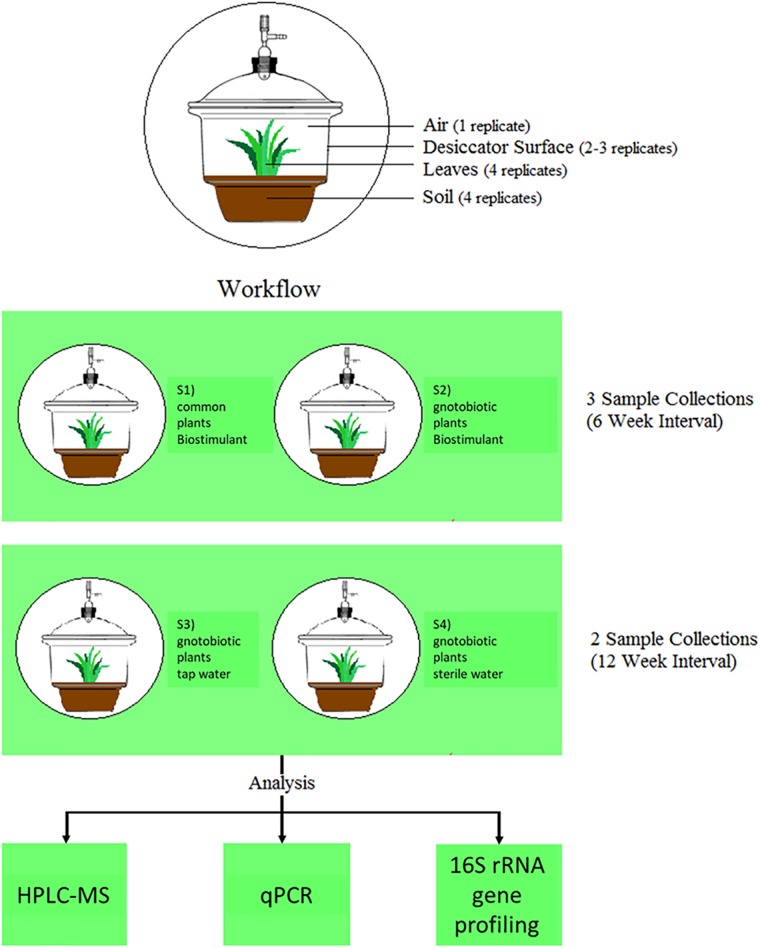
Experimental setup and workflow of this study.

In the frame of this study we applied the model biostimulant “bio-guss universal compost tea” (GARTENleben GmbH, Austria) on the house plant *Chlorophytum comosum* (Thunb.) Jacques (spider plant), a common indoor plant in homes and offices around the world, which showed meliorations of indoor air (Sriprapat et al., 2014) and the potential to change microbial diversity and abundance in its surroundings (Mahnert et al., 2015). The applied biostimulant consists of dried organic compost soil and plant residues in the form of tea-bags, which can be used for steeping plant irrigation waters and should act as a natural fertilizer with its own set of natural microorganisms^1^. The main ingredient is vermicompost produced by earthworms, which is a humus-like, nutrient and microorganism-rich compost (Lim et al., 2015). We developed a specific experimental design to test the effect of the biostimulant (Figure 1), and analyzed comparatively the microbiome by 16S rRNA gene profiling (diversity), qPCR (abundance), and HPLC-MS (metabolite profiling) to understand changes in microbial diversity and abundance in plant soil, on plant leaves and surrounding abiotic surfaces in the presence of the biostimulant its microbiota and metabolites.

## Materials and Methods

### Experimental and Sampling Design

For the experimental set-up 4 different plant systems were established (see Figure 1). Three of them contained plants, which were grown under gnotobiotic conditions and one was grown with natural seeds and soil. The biostimulant was added to the common plants and to one of the gnotobiotic plants. The remaining two functioned as control systems, which were irrigated with tab- and sterile water. All plants were grown for 12 weeks in a desiccator to minimize environmental influences and prevent microbial contaminations. The desiccators (Bartelt GmbH, Graz, Austria) were cleaned with water and detergent (Shower Cleaner, Bluestar, Germany), dried at room temperature and then closed to avoid further contamination. Afterwards they were dry-heated at 170°C for 24 h for sterilization and to degrade any remaining DNA. All sampling devices made from glass or metal (e.g., Erlenmeyer flasks, spatulas, tweezers etc.) and alpha-wipes (TX1009 Alpha-Wipe, ITW Texwipe, VWR, Austria) were heat-sterilized for 24 h at 170°C to degrade also DNA contaminants. The remaining plastic devices (e.g., 50 ml tubes) were UV-sterilized for 20–30 min under a laminar flow. All sampling devices were sterilized again before each sample collection.

### Propagation of Seeds and Plants

Four different desiccator systems (incubation chambers) were prepared according to Figure 1. The whole set-up comprised three plants, which were grown from surface-sterilized seeds in autoclaved soil and one plant grown under natural conditions. The gnotobiotic plants were watered with the biostimulant, tap water or sterile ultrapure water. The plants growing in natural soil were only treated with the biostimulant. Seeds of *Chlorophytum comosum* were provided by the botanical garden of Graz. For the gnotobiotic cultivation, the soil (Profi-Substrat, Gramoflor, Vechta, Germany) was autoclaved two times in an interval of 3 days and the seeds were surface sterilized using 2% sodium hypochlorite solution (Carl Roth GmbH & Co. KG, Karlsruhe, Germany) for 5 min. Afterwards the seeds were rinsed six times with sterile dH_2_O. The natural soil and seeds remained untreated. The pre-growth of the gnotobiotic *C. comosum* plants started on September 23rd, 2016 and the natural seeds were sown on September 26th, 2016 in tiny plastic boxes (9 cm × 10 cm^2^). The gnotobiotic plants were transferred to bigger plastic boxes (15 cm × 15 cm) on December 12th, 2016. During the whole procedure, from pre-growth to the end of the experiment, the plants grew inside the greenhouse. Only for sample collection and aeration purposes, the plants were transferred to a clean bench for 1–3 h. The growth conditions inside the greenhouse (Binder KBWF 720, Tullingen, Germany) were set to 22°C with a day–night regime of 12:12 h (7 kLux).

### Plant Treatments

The irrigation of the plants began after an air sampling in the frame of the first sample collection. In addition, the desiccators were opened once a week for an hour under laminar flow to further decrease the high moisture content, which promoted fungal growth. The plants were watered in an interval of 2 weeks with a volume of 50–100 ml. The biostimulant solution was prepared according to manual instructions^2^. The biostimulant contained vermicompost, malt sprouts, stone dust and organic herbs (stinging nettle, comfrey, field horsetail, valerian, marigold) and the following nutrients: 1.8 mg/l NH4-N, 22 mg/l NO3-N, 230 mg/l N Kjeldahl., 11.3 mg/l PO4-P, 290 mg/l K, 650 mg/l TOC. One tea bag (45 ml, 36.59 g ± 0.22 g) filled with the dried biostimulant was added to 2 L of autoclaved ultrapure water (BioScience-Grade, nuclease-free, autoclaved, DEPC-treated water, Carl Roth GmbH & Co. KG, Karlsruhe, Germany), under a laminar flow. The flask was closed, and the suspension was steeped for 24 h at room temperature before the tea bag was discarded. For each sampling event a new suspension of the biostimulant was prepared. The tap water was filled into sterile DNA-free glass ware and stored with the steeped biostimulant at 4°C in between the individual watering periods. The biostimulant (plant growth promoting agent “bio-guss universal compost-tea”) was applied as a liquid once a week over a duration of 12 weeks (3 months) on gnotobiotic plants grown in sterile and common soil. Plants watered only with tap water or sterile water were used as a control. By the use of sterile tubes implemented through the faucet of the desiccator and by placing the opening of each tube directly (∼1 cm distance) above an uncovered region (no covering plant leaves) of plant soil in each system, spilling of irrigation solutions on abiotic surfaces or plant leaves was prevented. Plants were transferred to incubation chambers to guarantee a constant microclimate with a mean temperature of 22.8°C, relative humidity of 79.3% and illumination of 5.6 kLux (Supplementary Figure S1) as determined with a data-logger (LOG 32 TH-PDF-data logger, DOSTMANN electronic GmBH, Germany and HD450: Datalogging Heavy Duty Light Meter, Extech, United States) over the whole course of the experiment. As high levels of relative humidity lead to mold formation, weekly aeration of the desiccators under a laminar flow were conducted. These aeration events are noticeable by respective repetitive drops in relative humidity (see Supplementary Figure S1).

### Sample Collections

Samplings (see (Mahnert et al., 2015) for further details) of the biostimulant (500 μl), plant soil (100 mg near the plant stem), plant leaves and surrounding abiotic surfaces at three defined points in time together with control samples at two points in time of the air in the incubation chamber (13 l/min for 10 min), irrigation liquids (500 μl of tap water and sterile water), lab devices, sampling equipment and used reagents summed up to 158 samples for qPCR and 16S rRNA gene amplicon sequencing. In addition, samples of the biostimulant steeped for 1 and 24 h were investigated with HPLC-MS (Q Exactive^TM^ Hybrid Quadrupole-Orbitrap^TM^ Mass Spectrometer, Thermo Fisher Scientific, United States) in four replicates (see scheme of the workflow in Figure 1).

For samplings of the desiccator surfaces, sterilized desiccators (height: 38 cm, diameter: 33 cm) were placed inside the clean bench and the lid was opened without pressuring the faucet. A sterile 50 ml tube, containing a sterile and dampened alpha-wipe, was carefully taken out with a sterile tweezer. In the first sample collection the whole inner surface of the desiccator was sampled with constant pressure, turning the wipe over after sampling half of the whole inner-surface of the desiccator. Directly after sampling, the alpha-wipe was transferred to an Erlenmeyer flask, containing 40 mL 0.9% sterile DNA-free sodium chloride solution. Contaminating the sampling wipe with plant soil or the transfer of it was prevented at following sampling events. This procedure was repeated for all four desiccators. For negative controls (field blanks), one wipe which was directly transferred from the falcon tube to the flask without touching any surfaces was processed in parallel. After the first sampling, the *C. comosum* plants were transferred to their specific desiccators. The leaves in each desiccator were counted and measured with a sterilized ruler. Each plant leaf sample was represented in four replicates. Each replicate covered a quarter of the leaves’ total surface area. Both sides of each leaf were sampled with constant pressure. Then, wipes were transferred into sterile Erlenmeyer flasks, which already contained 40 ml sterile, DNA-free 0.9% sodium chloride solution. Field blanks were processed as indicated above. With a sterile spatula, 100 mg of soil near the stem was transferred directly to a Lysing Matrix E Tube from the FastDNA Spin Kit for Soil in four replications. Subsequently the air from desiccators treated with the biostimulant was sampled with the SKC BioSampler (SKC Inc., PA, United States). All parts of the air sampler were autoclaved and dry-heated to achieve sterility and to degrade DNA. For the air sampling setup, the vacuum pump was connected to the desiccator over the faucet opening and to a beaker containing 10 ml PCR-grade water by sterile tubes. After the setup was installed, the vacuum pump was set to sample 13 l of air for 10 min. Sampling events were concluded with irrigating the plants with respective treatment types (biostimulant, tab-water or sterile water). An aliquot of 500 μl of each treatment was analyzed at each sample collection. Distances between the plant leaf surface and surrounding surfaces of the desiccator decreased from a maximum of 14 cm at the beginning to a minimum of 1 cm till the end of the experiment.

### DNA Extraction

Samples from wipes and the air were concentrated by repeated centrifugation cycles at 3220 × *g* and 4°C for 5 min with filter tubes (Amicon Ultra-15 Centrifugal Filter Units, Merck, Germany) to 500 μl, after vortexing for 10 s and sonication at 40 kHz for 2 min in an ultrasonic bath (Transonic Digitals, Elma, United States). PMA treatment and light crosslinking was performed as described in (Moissl-Eichinger et al., 2015). Concentrated and treated cell suspensions were then homogenized with a FastPrep-24 Classic Instrument (MP Biomedicals, United States) 2× for 30 s at 6.5 m/s. Tubes were cooled on ice for 30 s between homogenization cycles. Then genomic DNA was extracted with the FastDNA^®^ Spin Kit for Soil (MP Biomedicals, Solon, OH, United States) according to manufacturer instructions.

### Quantitative PCR (qPCR)

Quantitative PCR was conducted with the primers 515f-(GTGCCAGCAGCCGC) and 927r-(CCCGTCAATTYMTTTGAGTT) (0.5 μl of 5 μM each) on a Rotorgene 6000 instrument (Celtic Diagnostics, South Africa). Beside primers, the 10 μl reaction mix contained 5 μl KAPA SYBR FAST qPCR Master Mix Universal, 3 μl PCR-grade water, and 1 μl template DNA. Amplifications were achieved through 40 cycles of denaturation at 95°C for 20 s, annealing at 54°C for 15 s and elongation at 72°C for 30 s. A melt curve from 72 to 95°C (5 s per 1°C increase) together with standards based on *Bacillus subtilis* B2G and no template controls served as quality controls for amplified products. qPCR runs with reaction efficiencies above 1.2 and *R*^2^-values above 0.99 were considered to be of sufficient quality to determine microbial abundance.

### 16S rRNA Gene Amplicon Libraries

Amplicons were generated by two separate PCR reactions on a TPersonal thermocycler (Biometra, Germany). In the first step pads were added together with the primers [0.3 μl of 515f-(GTGYCAGCMGCCGCGGTAA)-pad and 926r-(CCGYCAATTYMTTTRAGTTT)-pad primers, 10 μM each] onto the target sequence in a 30 μl PCR reaction mix with 22.4 μl PCR-grade water, 6 μl Taq & Go PCR master mix and 1 μl template DNA. PCR products were amplified by 30 cycles of denaturation at 95°C for 45 s, annealing at 55°C for 45 s, and elongation at 72°C for 90 s. The PCR was repeated three times for each sample and 20 μL from each PCR reaction was pooled and used as template for the second PCR. In the second step, individual barcodes were attached to the pads in a 50 μl PCR reaction mix (details see above) and 15 cycles of denaturation at 95°C, annealing at 53°C and elongation at 72°C for 30 s at each step. The second PCR step was repeated four times for each sample and checked for quality by gel electrophoresis. Pooled PCR products were purified with the Wizard SV Gel and PCR clean-up System kit (Promega, United States) before they were quantified with a NanoDrop UV-Vis instrument (Thermo Scientific, United States). 50 μg DNA of an equimolar concentrated amplicon pool was then sent to the GATC Biotech AG, (Konstanz, Germany) for Illumina HiSeq sequencing using an optimized protocol (Schwendner et al., 2017) to achieve 300 bp paired end reads in the rapid run mode after entry quality control and adapter ligation. Raw reads were deposited in the European Nucleotide Archive - ENA^3^ under project ID PRJEB27998.

### Bioinformatics and Statistics

Amplicon sequences were pre-processed in QIIME 1.9.1 (Caporaso et al., 2010) and analyzed with QIIME2 (versions 2017.10 – 2018.8) (Bolyen et al., 2018). Forward and reverse amplicon sequences were stitched with an overlap of 100 bp and redundant sequences were removed. Reads were imported into QIIME 2 and demultiplexed according to sample specific barcodes. Sequences were filtered and denoised into features with DADA2 (Callahan et al., 2016). Resulting feature tables were rarefied for a core diversity analysis including phylogenetic metrics (unifrac) of the alpha and beta diversity with default settings. Representative sequences were aligned and filtered with mafft (Katoh and Standley, 2013) before a phylogenetic tree was calculated and rooted with fasttree (Price et al., 2010). Kruskal–Wallis tests (Kruskal and Wallis, 1952) were calculated for alpha diversity metrics to define significance between categorical metadata columns. Likewise, PERMANOVA tests (Anderson, 2001) based on 999 permutations were executed to define significance for beta diversity metrics between categorical metadata. For numerical metadata columns, significant correlations were determined by Spearman rank correlations and mantel tests (Pearson, 1895; Spearman, 1904; Mantel, 1967). Community compositions were linked to environmental variables by bioenv tests (Clarke and Ainsworth, 1993). Taxonomic assignments of representative sequences were conducted through a naïve-bayes classifier (Pedregosa et al., 2012) trained on 16S rRNA gene OTUs clustered at 99% similarities within the Silva123 database release. Differential abundance of taxa was determined by ANCOM (Mandal et al., 2015) and gneiss (Morton et al., 2017). Longitudinal analysis (Bokulich et al., 2018a) were based on changes in Shannon diversity estimates and weighted unifrac distances using linear mixed effects modeling (Seabold and Perktold, 2010) and non-parametric microbial interdependence tests (Zhang et al., 2017). Sample metadata was predicted with supervised machine learning classification and regression methods (Bokulich et al., 2018b) and maturity index prediction (Subramanian et al., 2014). Predictions of potential functional capabilities and contributions of distinct ASVs to particular functions were executed in PICRUSt (Langille et al., 2013). BugBase (Ward et al., 2017) was used to predict potential microbial phenotypes and SourceTracker (Knights et al., 2011) was applied to estimate the potential origin of characteristic taxa. Two-way ANOVAs were calculated in R (R Core Team, 2014) to determine any groupings of the qPCR data by the factors treatment and time.

### Metabolite Profiling

Metabolites of the biostimulant were analyzed by high-performance liquid chromatography (HPLC) coupled to mass spectrometry (MS). One bag of the biostimulant was transferred to 100 mL autoclaved water in four replicates. Two bags were steeped for 1 h and two were steeped for 24 h at room temperature. Afterwards respective bags of the biostimulant were carefully removed and 1 ml of the solutions were centrifuged at 13,500 × *g* and 4°C for 10 min. The resulting supernatants were analyzed with a HPLC-MS (Q Exactive^TM^ Hybrid Quadrupole-Orbitrap^TM^ Mass Spectrometer, Thermo Fisher Scientific, United States) on an Atlantis column at 0.3 mL/min including a gradient of 40% of Component B for 2 min – 100% of Component B for 15 min – and 40% of Component B for 5 min, and a run voltage of 3100 V and a capillary temperature of 330°C for 40 min. Component A contained 0.1% formic acid in double distilled H_2_O, while Component B contained 0.1% formic acid solved in acetonitrile. The water, used for steeping, served as a blank. Positive and negative mode were separately executed with a resolution of 70.000, an AGC target of 10^6^, Maximum IT set to 200 ms, a scan range of 100–1500 mass to charge ratios and a resolution of 17.500 for the MS2-Parameter. Metabolite analysis was performed with Compound Discoverer 2.1 (Thermo Fisher Scientific). Spectra were compared with database entries on mzCloud^4^ and simulated spectra from CFM-ID (Allen et al., 2014).

## Results

### Impact on Plant Growth

In the course of the experiment the leaf area increased in all systems (Supplementary Figure S2). The highest increase was evident for common plants irrigated with the biostimulant (8-fold), followed by gnotobiotic plants irrigated with the biostimulant (4-fold) and sterile water (1.5-fold). Only gnotobiotic plants irrigated with tap water showed a decline in plant growth from 0.17 to 0.16 m^2^. The plants which were treated with the biostimulant (common and gnotobiotic plants) did not show a significantly higher growth compared to the other systems (two-way ANOVA: *P* = 0.075; Supplementary Table S1). However, the plants started with different leaf areas, which makes comparisons even less objective.

### Impact on Microbial Abundance

**FIGURE 2:**
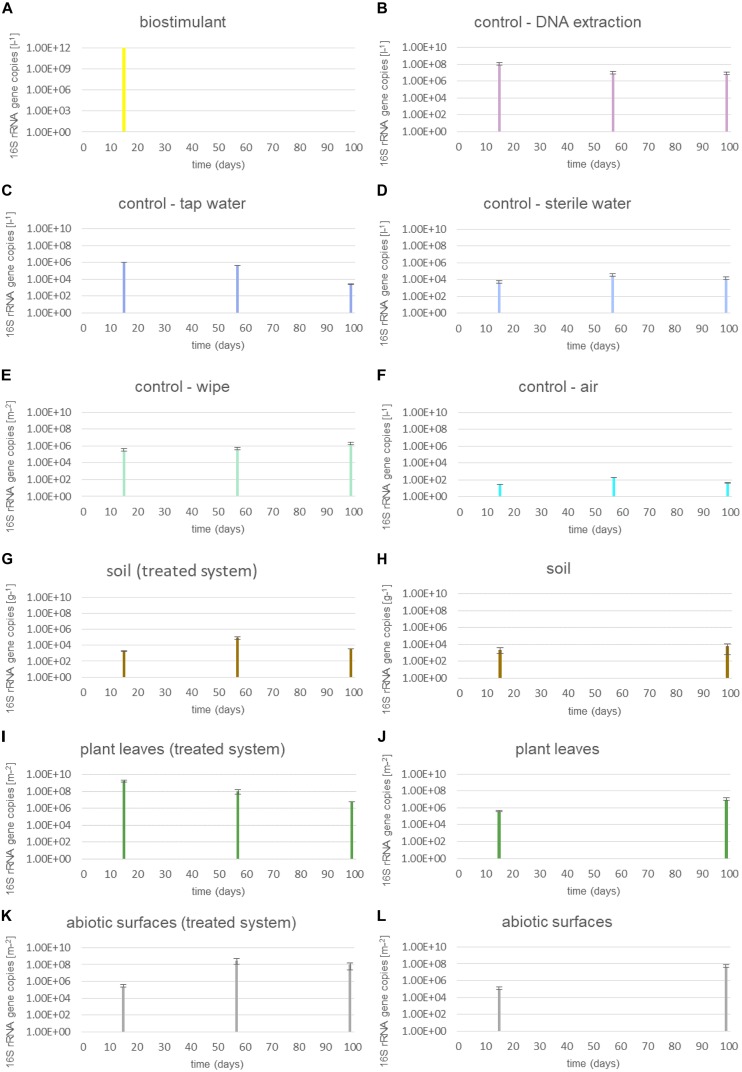
Microbial abundance of samples treated with the biostimulant and untreated samples. **(A)** Steeped biostimulant, **(B)** control of DNA extraction reagents, **(C)** tap water control, **(D)** sterile water control, **(E)** wipe control, **(F)** air control, **(G)** soil of biostimulant treated systems, **(H)** soil of systems without contact to the biostimulant, **(I)** plant leaves of biostimulant treated systems, **(J)** plant leaves of systems without contact to the biostimulant, **(K)** desiccator surfaces of biostimulant treated systems, and **(L)** desiccator surfaces of systems without contact to the biostimulant. 16S rRNA gene copy numbers were extrapolated to 1l steeped biostimulant **(A)**, DNA extraction reagents **(B)**, irrigation waters **(C,D)**, or air **(F)**, 1 g soil **(G,H)**, 1 m^2^ wipe **(E)**, plant leaf area **(I,J)** or desiccator surface **(K,L)**.

Microbial abundance of dry and dissolved biostimulant was very high (∼10^12^ 16S rRNA gene copies per gram or liter). The microbial abundance increased in the soil of the plant (∼10^3^ to ∼10^9^ 16S rRNA gene copies per g) and on surrounding abiotic surfaces (∼10^5^ to ∼10^7^ 16S rRNA gene copies per m^2^), while samples from plant leaves (∼10^8^ to ∼10^6^ 16S rRNA gene copies per m^2^) and controls showed a decrease in microbial abundance (Figure 2). A two-way ANOVA revealed a significant grouping of microbial abundances for plant leaves and surrounding abiotic surfaces for the factor time, but no significance for the type of treatment or an interaction of both factors (treatment and time; Supplementary Table S2).

The proportion of 16S rRNA gene copy numbers from intact microbial cells was determined through a treatment with the chemical PMA prior to DNA extraction procedures. This differentiated analysis showed that the dissolved biostimulant contained mainly intact microbial cells (up to 91%, 1.2 × 10^11^ 16S rRNA gene copies per liter; Supplementary Figure S3). On plant leaves the proportion of intact microbial cells was lower compared to the dissolved biostimulant. Interestingly, plant leaves treated with the biostimulant showed a higher proportion of intact cells (42%) compared to plants irrigated with tap or sterile water (20%). Nevertheless, any groupings by these factors were not significant (Supplementary Table S3).

### Impact on Microbial Diversity

The 16S rRNA amplicon library resulted in a total of 54,330,655 sequences (forward and reverse, respectively) with a length of 301 bp. After filtering and denoising 158 samples contained on average 10,551 ASV (amplicon sequence variants; minimum 2, maximum 552,293) and 13,405,388 reads per sample (minimum 2, maximum 242,282).

**FIGURE 3:**
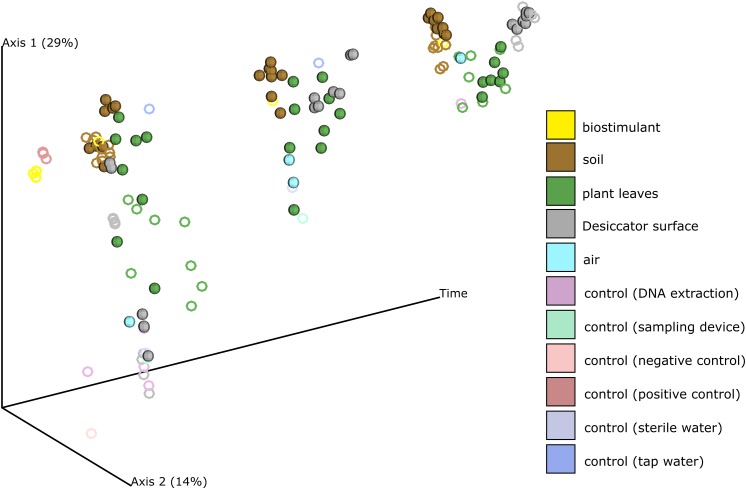
PCoA of the changing microbial composition over time and after application of the biostimulant. Desiccator surfaces experienced the biggest shift in their microbial composition and became more leaf-like. Samples from biostimulant treated test systems are shown as spheres and samples from test systems treated with sterile and tap water are shown as rings.

Beside changes in microbial abundance we could also determine distinct changes of microbial composition on plant leaves and on surrounding abiotic surfaces of the plant (Figure 3, Supplementary Figures S4, S5 and Supplementary Tables S4, S5). While samples from abiotic surfaces were similar to controls at the beginning of the experiment, their composition changed together with those from plant leaves along PCoA Axis 1 till the end of the experiment (Supplementary Figure S6). During the experiment not only plant leaves became more similar to each other, also samples from abiotic surfaces became more leaf-like. Clustering of samples according to their type of treatment was minor and not significant in pairwise comparisons (Supplementary Figures S7, S8 and Supplementary Table S5).

**FIGURE 4:**
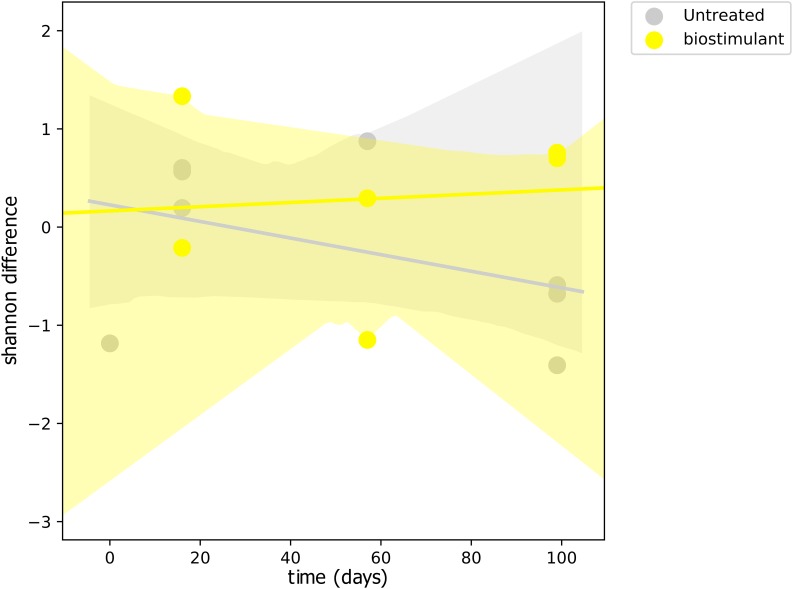
Regression scatterplot to track the rate of change in microbial diversity (Shannon *H*′) from a baseline through the course of the experiment for samples treated with the biostimulant and untreated samples.

**FIGURE 5:**
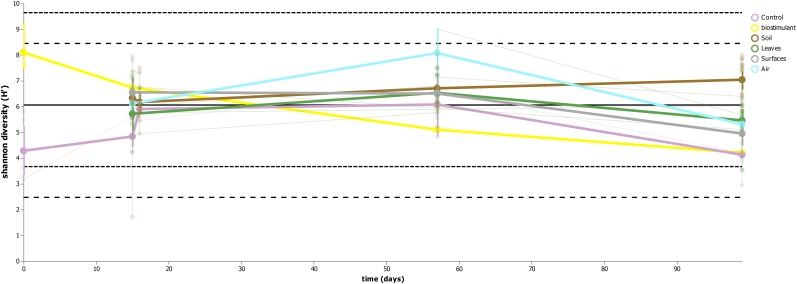
Volatility plot indicating changes of microbial diversity (Shannon *H*′) for the main sample categories (controls, biostimulant, soil, plant leaves, desiccator surfaces and air).

**FIGURE 6:**
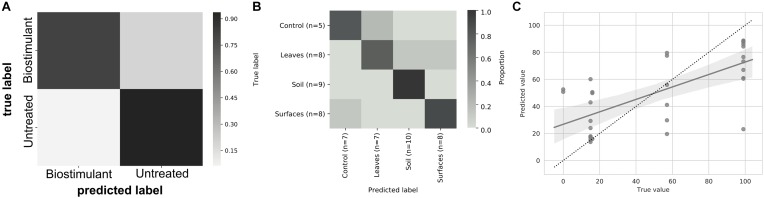
Heatmap of the prediction ability of samples treated with the biostimulant **(A)**, the main sampling categories air, controls, plant leaves, soil, and desiccator surfaces **(B)**, and the day of sampling **(C)**. Machine learning tools based on random Forest classification and regression models were used to train the software to predict a certain metadata category from its ASVs (amplicon sequence variants) profile.

This process was accompanied by a decrease of microbial diversity (Shannon diversity *H*′ in the air: 6.1–5.3, controls: 5.0–3.6, surrounding abiotic surfaces: 6.6–5.0, plant leaves: 5.7–5.5, biostimulant: 7.8–4.2). Only soil samples showed an increase of microbial diversity during the course of the incubation period (*H*′: 6.2–7.0). However, the overall decrease of microbial diversity was much lower or even impeded for those samples which were treated with the biostimulant (in direct or indirect contact with the biostimulant: surrounding abiotic surfaces 6.8–5.3, plant leaves 5.5–5.4, soil 6.4–7.3; non-treated samples: surrounding abiotic surfaces 6.3–4.6, plant leaves 6.2–5.4, soil 5.8–6.8; see Figures 4, 5). Nevertheless, pairwise difference tests based on a Wilcoxon signed-rank test were not significant (Supplementary Table S6). Changes of Shannon diversity were accompanied by a decrease of richness, phylogenetic diversity and evenness for almost all sample types. Only samples of the plant soil experienced an increase of these alpha diversity metrics (Supplementary Figure S9). The microbial composition of samples was distinct enough to predict a treatment with the biostimulant to an accuracy of 87.5% with random Forest classification and regression models (see Figure 6A). Likewise, the origin of samples could be easily predicted for soil samples (100%) and to a lesser extent for surrounding abiotic surfaces (87.5%) and controls (80%). Plant leaves were not that characteristic to predict microbial compositions (75%) (Figure 6B) as well as the day of sampling (*R* = 0.68, *P* = 1.7 ^∗^ 10^-5^) (Figure 6C).

### Impact on Microbial Composition

**FIGURE 7:**
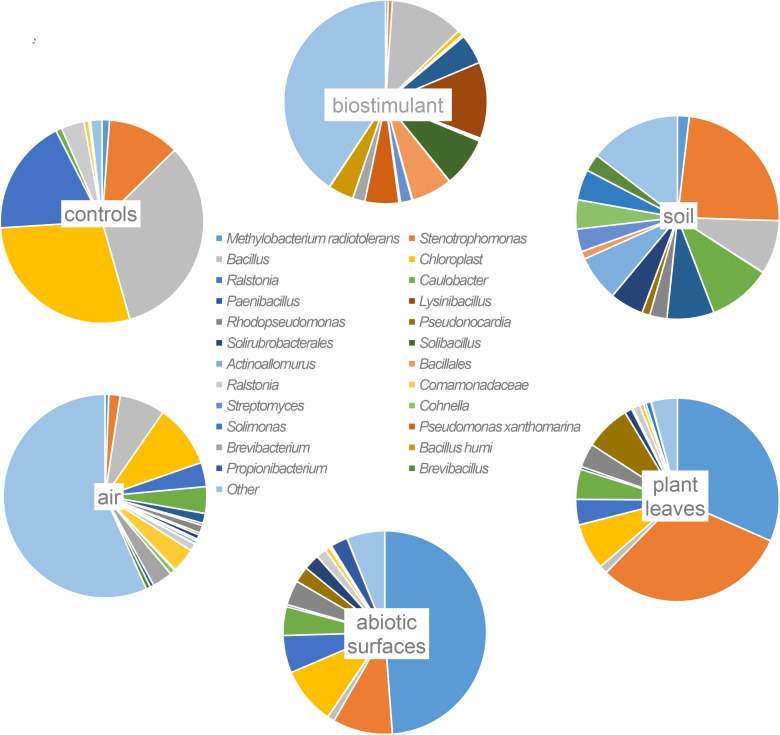
Most abundant taxa (>10% relative abundance) on highest taxonomic levels per origin of samples (abiotic surfaces = desiccator surfaces).

**FIGURE 8:**
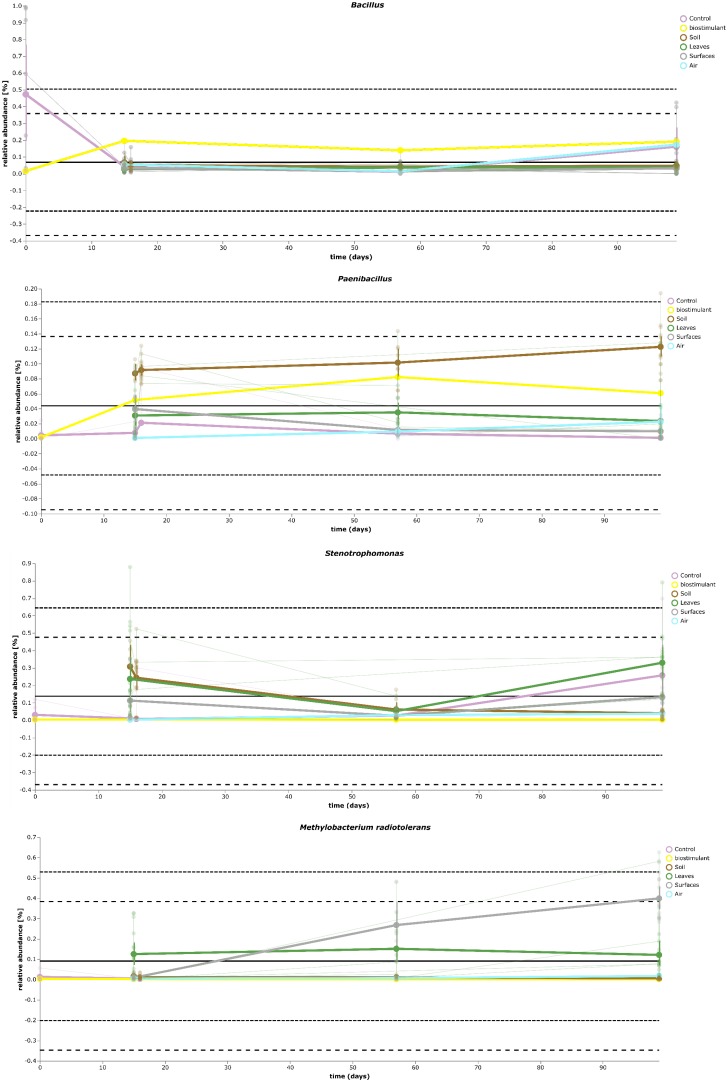
Volatility plot of selected taxa on genus and species level showing distinct changes in relative abundance for different type of samples (biostimulant – *Bacillus*, soil – *Paenibacillus*, plant leaves – *Stenotrophomonas*, desiccator surfaces – *Methylobacterium radiotolerans*).

**FIGURE 9:**
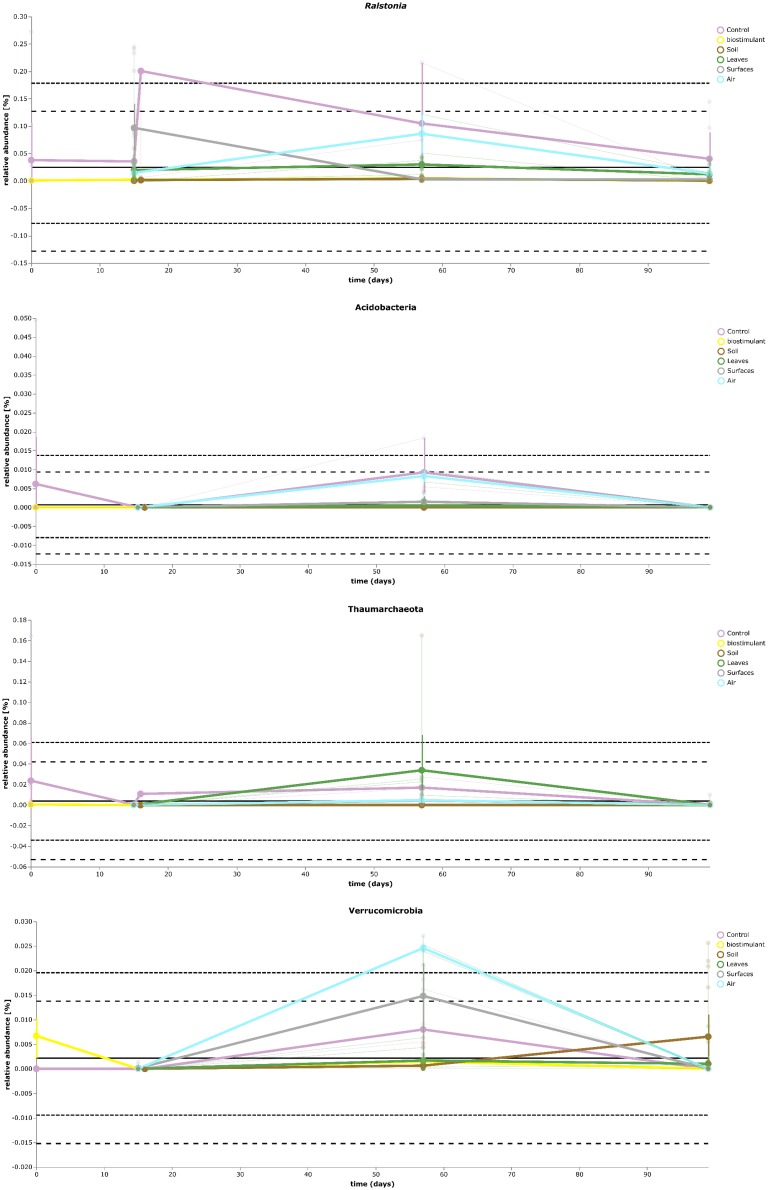
Volatility plot of selected taxa on genus and species level showing distinct changes in relative abundance for different type of samples (controls – *Ralstonia*) and of the transient occurrence of new phyla (*Acidobacteria, Thaumarchaeota*, and *Verrucomicrobia*).

**FIGURE 10:**
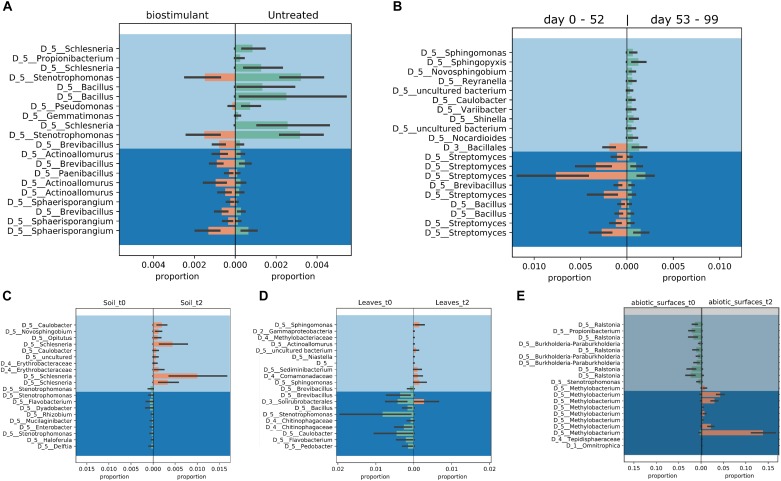
Proportion plots of differential abundance analysis using balances in gneiss according to type of treatment **(A)**, time of sampling **(B)**, soil **(C)**, plant leaves **(D)**, and samples of the desiccator surfaces **(E)**.

Most ASVs were assigned to the phyla *Proteobacteria, Firmicutes*, and *Actinobacteria*. Compared to gnotobiotic plants, leaves and soil of common plants showed higher relative abundances of *Bacteroidetes*. Further differences on phylum level were apparent between the dry and dissolved biostimulant. In contrast to its dry counterpart the steeped biostimulant showed a higher relative abundance of *Firmicutes* (dissolved biostimulant 76%, dry biostimulant 17%), while all other assigned phyla showed a lower relative abundance in comparison to the dry biostimulant. At the end of the experiment, *Proteobacteria* was the most abundant microbial phylum with an average of 80.1 ± 12.1% on plant leaves and on surrounding abiotic surfaces. In contrast, soil samples showed a balanced distribution of *Firmicutes, Proteobacteria*, and *Actinobacteria*. On genus and species level (see Figure 7) most sequences could be assigned to *Methylobacterium radiotolerans* (up to 75%), *Stenotrophomonas* sp. (up to 63%), *Lysinibacillus* sp. (up to 20%), *Bacillus* sp. (up to 19%), *Caulobacter* sp. (15%) and *Paenibacillus* sp. (11%). While *Caulobacter* and *Paenibacillus* were typically observed in samples of the plant soil, the biostimulant was rich in signatures of *Lysinibacillus* and *Bacillus*. During the experiment a predominance of *Stenotrophomonas* formed on plant leaves and *Methylobacterium radiotolerans* dominated on surrounding abiotic surfaces (Figure 8). Noteworthy, new phyla, e.g., *Verrucomicrobia* (air and surfaces), *Acidobacteria* (air and controls) and *Thaumarchaeota* (plant leaves) manifest after the treatment with the biostimulant (Figure 9). Compared to real biological samples, controls and technical samples showed other lineages like *Ralstonia* (Figures 7, 9) for tap and sterile water or sequences of *Propionibacterium* on alpha wipes. Differential abundance analysis using balances in gneiss revealed that proportions of *Paenibacillus, Brevibacillus, Actinoallomurus*, and *Sphaerisporangium* were higher in samples and test systems treated with the biostimulant (Figure 10A). During the whole incubation period the proportion of sequences assigned to *Caulobacter, Novosphingobium*, or *Schlesneria* increased in particular in the plant soil (Figures 10B,C), *Sphingomonas* on plant leaves (Figure 10D) and *Methylobacterium* on the surrounding surfaces of the desiccator (Figure 10E). Most of these bacterial genera are typical representatives of plant-associated microorganisms, living in the phyllosphere and rhizosphere of plants (*Methylobacterium radiotolerans, Rhodopseudomonas*) or on abiotic surfaces (*Stenotrophomonas*) and water-bearing environments (*Caulobacter*). A core microbiome analysis showed seven ASVs, which were shared to proportions of 80 and 90% with all samples. For samples of the biostimulant and from soil, shared ASVs (80%) were assigned to *Bacillus humi* and *Janibacter*. *Stenotrophomonas*, and *Methylobacterium radiotolerans* were shared (80%) between samples from the biostimulant, plant leaves and surrounding surfaces of the desiccator. Likewise samples from soil, plant leaves and desiccator surfaces as well as samples from plant leaves and desiccator surfaces, respectively, shared (90%) the same ASV assigned to *Methylobacterium radiotolerans*.

**FIGURE 11:**
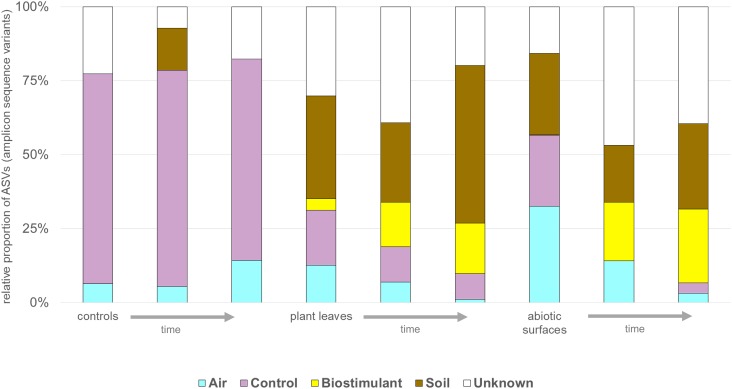
The potential sources of microbes determined with SourceTracker. Change in proportions [%] of microbes from air, controls, the biostimulant, soil and unknown sources for control, plant leaf and desiccator surfaces (abiotic surfaces) samples over time (indicated by an arrow).

**FIGURE 12:**
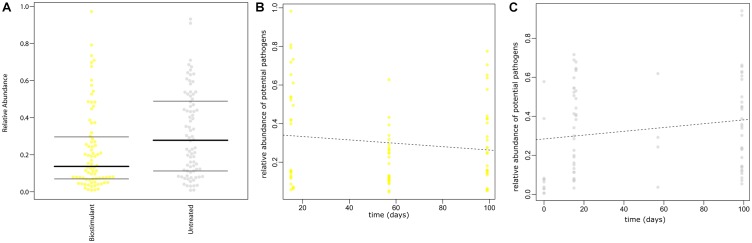
**(A)** Prediction of potential pathogenic phenotypes for treated samples with the biostimulant and untreated samples. **(B)** Prediction of potential pathogenic phenotypes over time for samples treated with the biostimulant and **(C)** for untreated samples. Predictions are based on the Greengenes database reference set clustered at 97% similarity and precalculated files created with the help of PICRUSt, IMG, KEGG and PATRIC. The plot shows the relative proportion [%] of potential pathogenic traits inferred from the 16S rRNA gene profile.

SourceTracker was used to identify potential sources of observed microbes (see Figure 11). At the beginning of the experiment plant leaf samples and samples from surrounding abiotic surfaces were almost void of typical microbial signatures of the biostimulant. However, during the incubation period, their proportion increased on plant leaves (4–17%) and even highly significant in the case of samples from surrounding abiotic surfaces (0–25%; two-way ANOVA *P* = 1.1 ^∗^ 10^-7^). Nevertheless, beside the factor time no significant increase could be determined for the type of treatment or an interaction of both factors (treatment and time; Supplementary Table S7). In addition, according to phenotype predictions with BugBase, significantly lower proportions of potential pathogens (Mann–Whitney–Wilcoxon Test, FDR-corrected *P* = 0.006, Supplementary Table S8) were detected in samples treated with the biostimulant (Figure 12A). Correlations of this decline in potential pathogens over time were not significant (Spearman’s rank correlation, *R*: -0.06, *P* = 0.6) for samples treated with the biostimulant, but the increase in potential pathogens over time was significant for untreated samples (Spearman’s rank correlation, *R*: 0.3, *P* = 0.003) (Figures 12B,C and Supplementary Tables S9, S10).

### Impact on the Metabolic Profile

HPLC-MS measurements of the steeped biostimulant after 1 and 24 h of incubation revealed several plant growth promoting substances (see Table 1). Each of the five most common compounds (except 4-amino-3-hydroxybenzoic acid) are important metabolites in biological degradation processes. The longer the incubation time, the more of the compounds dissolved in the suspension (e.g., 4-amino-3-hydroxybenzoic acid increased 13-fold after 24 h). However, no new compounds were found after 24 h compared to 1 h of steeping the biostimulant.

**Table 1:** High-performance liquid chromatography (HPLC)-MS results for the steeped biostimulant.

Name	Sample/control ratio	KEGG pathways	Mean area (1 h)	Mean area (24 h)	Difference 1 vs. 24 h [%]
Indole-3-acetic acid	4.8	Metabolic pathways, Biosynthesis of plant hormones and alkaloids derived from terpenoid and polyketide, Plant hormone signal transduction, Tryptophan metabolism	607016.2	2666757.8	439.3
Syringic acid	2.3	Microbial metabolism in diverse environments, Aminobenzoatedegradation	731223.7	1649582.1	225.6
4-Amino-3- Hydroxybenzoic acid	13.4	–^∗^	42164.6	589748.7	1398.7
2-Naphthalene sulfonic acid	15.1	Naphthalene degradation	49270.5	319698.5	648.9
Acetophenone	3.4	Microbial metabolism in diverse environments, Ethylbenzene-, DDT-, Bisphenol- and aromatic compounds degradation	99621.9	212796.9	213.6


*^∗^No KEGG pathway entry found.*

*Sterile H_2_O was used for steeping the biostimulant and served as a control. The five most abundant compounds are listed, which showed a sample to control ratio over 2. Detected metabolites were set into the context of KEGG pathways (http://www.genome.jp/kegg/pathway.html) to identify their potential role in biological systems.*

We used PICRUSt to predict functional capabilities from detected microbial compositions (Supplementary Table S11) and determine the contributions of certain taxa to detected metabolites of the HPLC-MS analysis. This co-observation of metabolites, predicted functions and potential microbial contributors revealed that species of *Pseudomonas* could have contributed to the detected metabolites in the biostimulant to proportions of up to 0.2%.

## Discussion

In this study we showed that biostimulants for plants applied to soil have a potential to shift the microbiota on the above-ground parts of the plant as well as in the surrounding. They supported especially ASVs with beneficial representatives and counteract the loss of microbial biodiversity. The obtained results support the idea that biostimulants can have a positive impact on plant growth and performance by shifting the microbiome and metabolome as well. This can explain the often-reported plant growth promoting effect for biostimulants. However, our experimental setup was not sufficient to significantly distinguish between effects from incubating conditions over time on the microbiome and actual effects of the biostimulant. In addition, the underlying mechanism of microbial transfer is still unknown. Since simple spilling of the irrigation solution was prevented, we suspect other vectors like microscopic aerosols, or actual microbial locomotion from the rhizosphere through the endosphere to the phyllosphere and microbial deposition from plant leaves to surrounding abiotic surfaces as most promising explanations of the observed phenomena. Our ideas are based on the reported transfer of microorganisms from other holobionts to their surroundings (Qian et al., 2012) and the obvious connection between different sampled microenvironments and microbiomes (Badri et al., 2013). Nevertheless, extensive analysis of the changing air quality as a main target for microbial transfer was not possible due to the detection limit of the applied methods and therefore this analysis was beyond the scope of this study.

At phylum level, the treatment resulted in comparison to the untreated control in an increase of *Bacteroidetes* and a surprising peak of new phyla at the second sampling event, e.g., *Verrucomicrobia, Acidobacteria*, and *Thaumarchaeota. Verrucomicrobia* comprise mainly as-yet uncultivated species; several have been already identified in association with plant hosts (Bragina et al., 2015). Species of the phylum *Acidobacteria* (e.g., *Granulicella paludicola, G. pectinivorans, G. aggregans, G. rosea, Acidicapsa borealis, A. ligni*, and *Terriglobus tenax*) were shown to actively interact with plants and act as plant growth-promoting bacteria (Kielak et al., 2016). Interestingly, plant hosts were shown to select particular groups of *Acidobacteria* and *Verrucomicrobia* (Da Rocha et al., 2013). Recently, representatives from *Thaumarchaeota* were identified to be also plant-associated with potential beneficial functions such as the production of phytohormones, which were identified by metagenomic mining (Taffner et al., 2018). Unfortunately isolates of all three phyla are difficult or even impossible to obtain. Therefore, the observation that these groups can be enriched by biostimulants is an important finding for targeted microbiome engineering. The microbiome shift was also visible at genus level; here the proportion of potentially beneficial microorganisms like *Methylobacterium, Stenotrophomonas*, and *Caulobacter* increased relatively and significantly for *Brevibacillus, Actinoallomurus, Paenibacillus*, and *Sphaerisporangium*. Species of *Brevibacillus* have potential to act as biological control agents as they were shown to produce chitinases to degrade fungal cell walls (Hassi et al., 2012) or can act pesticidal against insects, nematodes and mollusks (Ruiu, 2013) and plant-associated endophytic Actinobacteria like *Actinoallomurus* and *Sphaerisporangium* were already suggested as plant-growth promoting agents in the past (Qin et al., 2011; Hamedi and Mohammadipanah, 2014). Furthermore, the overall loss of microbial diversity was reduced, which took place under our experimental conditions, and the built environment became more plant-like.

Our model biostimulant is a mixture containing dried vermicompost, compost and plant residues. These mixtures are typical for biostimulants and therefore it is often difficult to identify the active ingredient. Results of our study showed that the biostimulant contain a high number of intact microbial cells, which definitely contribute to the microbiome shift and effect. Obviously, the positive effect of the biostimulant was not derived from its microbial content alone. The metabolites, which were determined in the biostimulant, contained properties, which can be useful for both the plants and the human’s well-being. For instance, syringic acid acts antifungal (Chong et al., 2012) and indole-3-acetic acid has plant growth promoting effects (Estelle, 2001). Moreover, acetophenone and syringic acid even have reported health benefits, with improvement against hypoglycemia and diabetes (Muthukumaran et al., 2013; Jiang et al., 2018). However, it was not investigated in this study, if these properties could be passed on to the benefit of human health. Hence, follow-up studies should extend our findings to human well-being, other plant species and biostimulants as well as experimental settings that better represent normal room conditions. Unfortunately the metabolome was not investigated at all sampling events in parallel with the microbiome. Moreover late stage effects of the microbiome and metabolome were not monitored. In addition, actual functional metagenomics of the treated microbiomes would be helpful to identify changed metabolic capabilities beyond simple correlations of predicted pathways based on static databases and measured metabolites.

We used an indoor plant as a model; all house plants have amazing capacities beyond simple embellishments, e.g., to improve indoor air quality (Sriprapat et al., 2014), and human performance in built environments (Bringslimark et al., 2009). As we could show before, plants can also shape the microbiome in an indoor environment (Mahnert et al., 2015). Therefore, indoor plants can act as vectors to transfer beneficial microbiota and increase biodiversity of microbial wastelands in built environments (Gibbons, 2016). Even more goal-driven is the application of a defined microbial consortia as a biostimulant on an indoor plant to manipulate the microbiome of an indoor environment. As we could show, beneficial properties of the biostimulant (high diversity of intact and beneficial microbiota) may be transferrable beyond the plant itself and extendable to the close vicinity of it. However, it is important to note that necessary compromises of the experimental set-up could limit general validity of our results. Most indoor environments are characterized by a few indoor plants in vast open spaces. Here, the proportion of plant-leaf to surrounding abiotic surfaces, the number of occupants their actions, the microclimate and longitudinal parameters can be completely different. In addition, effects of the substrate were pretty much ignored. The rhizosphere of common soil is a hotspot for diverse microbial interactions, which are absent in the sterilized soil we applied. Furthermore the impact of fertilizers and nutrients was not investigated. Therefore future studies should also include additional control experiments with synthetic nutrient mixtures, common soil with an active rhizosphere as well as non-soil based substrates. This would help to differentiate between actual drivers (nutrients, metabolites, or microbes) of observed microbial changes. Nevertheless, our experimental set-up tried to limit unknown influences by environmental parameters and establish sterile replicable test systems with defined settings by the use and comparison of sterile, DNA-free sampling equipment, plant soil, gnotobiotic plants, a constant microclimate and many controls of the environment. Apart from these limitations, our results indicate that core and shared microbial signatures of the biostimulant (e.g., *Methylobacterium*) were transferred from the irrigated soil not only to the plant surface itself, but also to surrounding abiotic surfaces. The detected microbiota may already support a healthy environment for the treated plant. For instance, *Lysinibacillus* and *Bacillus* function as bio-insecticides (Berry, 2012), and *Bacillus a*nd *Paenibacillus* augment plant growth (Bloemberg and Lugtenberg, 2001).

Hence, we envision combined effects of biostimulants not only for plant, but also human health in the future. Both plants as well as human beings rely on beneficial microbiota and overall diversity (Berg et al., 2017). However, common human activities in the environment reduce microbial diversity and therefore destabilize important microbial networks (Blaser, 2016; van der Heijden and Hartmann, 2016). This processes could facilitate the entry and establishment of pathogens in a system with serious consequences for the plant or human holobiont (Kennedy et al., 2002). As a proof of principle this study should be a first step to design biostimulants not only for plants, but also other holobionts in respective target environments in the future.

## Data Availability

16S rRNA gene amplicon raw data is available at the European Nucleotide Archive - ENA (https://www.ebi.ac.uk) under project ID PRJEB27998.

## Author Contributions

AM and GB study design. MS plant propagations. MH conducting experiments and measurements. AM and MH data analysis. AM, MH, and GB wrote the manuscript. All authors approved the final manuscript.

## Conflict of Interest Statement

The product “bio-guss universal compost tea” was provided by the GARTENleben GmbH. The company suggested the application of this product as a biostimulant in this study.

## Footnotes

1 http://www.gartenleben.at/bioguss

2 http://www.gartenleben.at/bioguss

3 https://www.ebi.ac.uk

4 https://www.mzcloud.org/
