# Supplementary material for: Enriching Beneficial Microbial Diversity of Indoor Plants and Their Surrounding Built Environment With Biostimulants
Source: Front Microbiol. 2018 Dec 5;9:2985. doi: 10.3389/fmicb.2018.02985 (PMC6290261; doi:10.3389/fmicb.2018.02985)
Supplement: Supplementary file 1 [file Data_Sheet_1.PDF]

Supplementary to:

Enriching beneficial microbial diversity of indoor plants and their surrounding built environment with biostimulants

Alexander Mahnert[1*], Marika Haratani[1], Maria Schmuck[1], Gabriele Berg[1]

[1]Institute of Environmental Biotechnology, Graz University of Technology, Graz, Austria

*Corresponding author:

Dr. Alexander Mahnert

alexander.mahnert@gmail.com

Target Journal: Frontiers in Microbiology, Research Topic: Biostimulants in Agriculture

Running title: Biostimulants for indoor plants in the built environment

Keywords: Indoor plants, built environment, biostimulants, vermicompost, microbiome, 16S rRNA gene amplicon analysis, qPCR, LC-MS

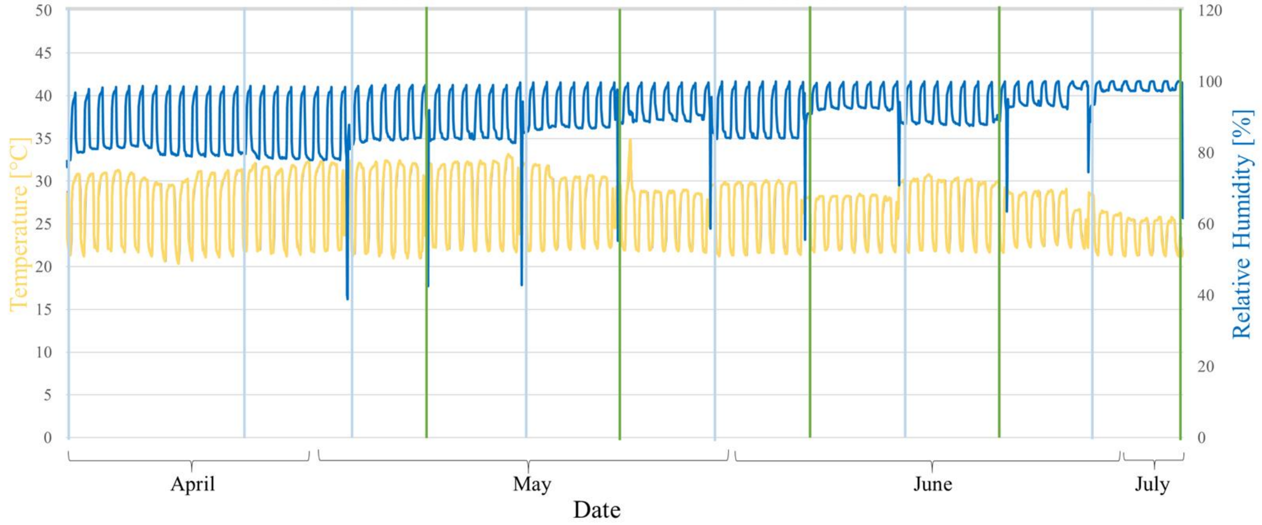

Supplementary Figure S1: Microclimate recordings inside desiccator 3. Blue line (relative humidity [%]). Yellow line (temperature [°C]). Vertical blue lines: desiccators were opened for an hour and watered, vertical green lines: desiccators were opened one hour under laminar flow

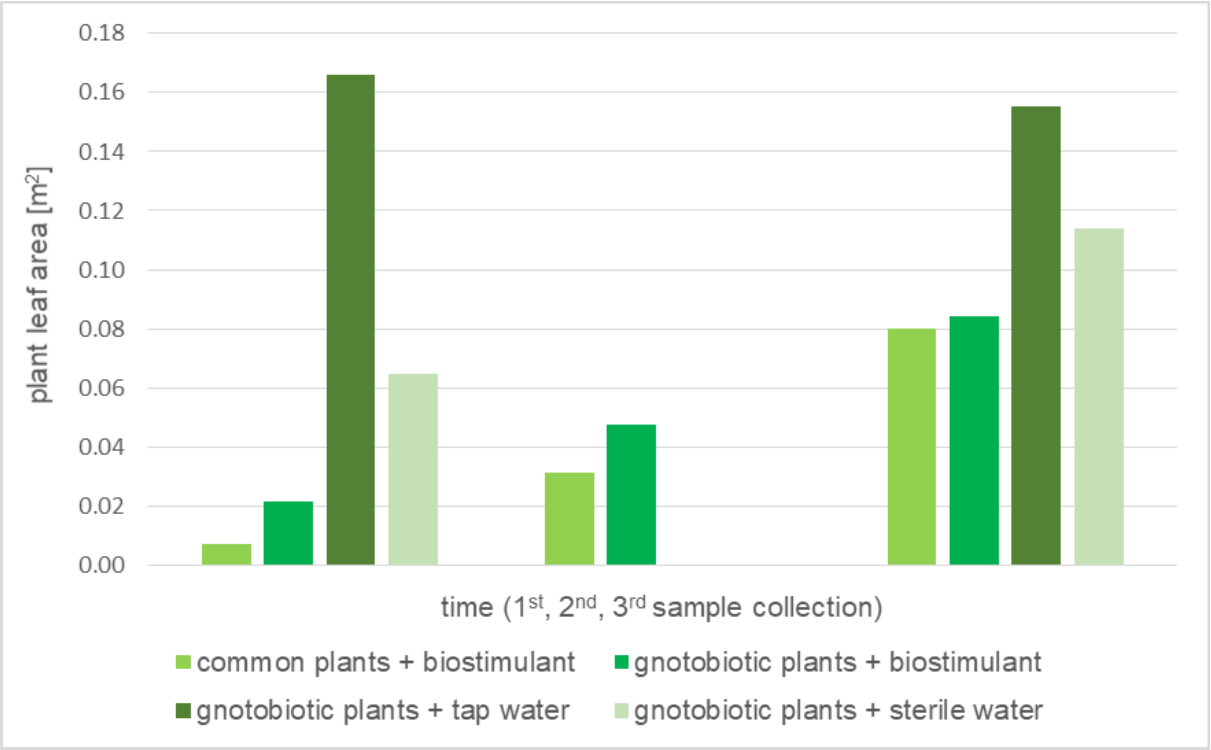

Supplementary Figure S2: Growth of plant leaves inside the different systems determined at three sampling events.

Supplementary Table S1: Two-way ANOVA results of the plant growth data (Figure S2).

ANOVA (plant growth)

Source of Variation	*SS*	*df*	*MS*	*F*	*P-value*	*Fcrit*
Sample	0.01125	1	0.01125	5.696203	0.07544	7.708647
Columns	0.0032	1	0.0032	1.620253	0.272007	7.708647
Interaction	0.00125	1	0.00125	0.632911	0.470831	7.708647
Within	0.0079	4	0.001975			
Total	0.0236	7				

Supplementary Table S2: Two-way ANOVA results of qPCR data from sample categories soil, plant leaves and desiccator surfaces.

ANOVA (soil)

Source of Variation	*SS*	*df*	*MS*	*F*	*P-value*	*Fcrit*
Sample	1.1915E+19	1	1.1915E+19	0.93179633	0.3426604	4.19597182
Columns	1.8428E+20	1	1.8428E+20	14.4113236	0.00072371	4.19597182
Interaction	1.1915E+19	1	1.1915E+19	0.93179566	0.34266057	4.19597182
Within	3.5804E+20	28	1.2787E+19			
Total	5.6615E+20	31				

ANOVA (plant leaves)

Source of Variation	*SS*	*df*	*MS*	*F*	*P-value*	*Fcrit*
Sample	6.2964E+18	1	6.2964E+18	3.81040916	0.06100107	4.19597182
Columns	6.1257E+18	1	6.1257E+18	3.7070942	0.06439359	4.19597182
Interaction	6.2233E+18	1	6.2233E+18	3.76612605	0.06242992	4.19597182
Within	4.6268E+19	28	1.6524E+18			
Total	6.4913E+19	31				

ANOVA (desiccator surfaces)

Source of Variation	*SS*	*df*	*MS*	*F*	*P-value*	*Fcrit*
Sample	6.4337E+15	1	6.4337E+15	2.27254334	0.14288458	4.19597182
Columns	3.4122E+16	1	3.4122E+16	12.0527426	0.0016967	4.19597182
Interaction	6.3854E+15	1	6.3854E+15	2.25550911	0.14433426	4.19597182
Within	7.9269E+16	28	2.831E+15			
Total	1.2621E+17	31				

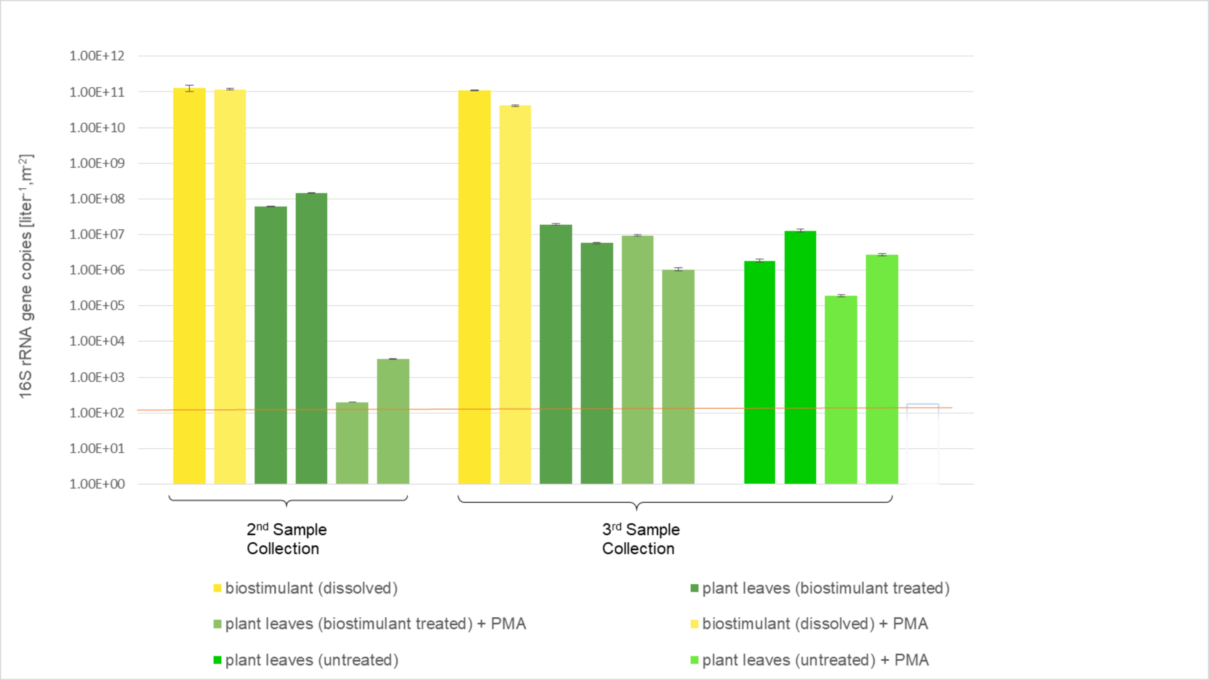

Supplementary Figure S3: Proportions of intact cells determined by PMA treatment of plant leaf samples and the biostimulant (dry and dissolved).

Supplementary Table S3: Two-way ANOVA on proportion of intact cells on plant leaves.

ANOVA (intact cells of treated plant leaves over time)

Source of Variation	*SS*	*df*	*MS*	*F*	*P-value*	*Fcrit*
Sample	6.0012E+15	1	6.0012E+15	6.72185853	0.06051438	7.70864742
Columns	3.5748E+15	1	3.5748E+15	4.00403949	0.11598275	7.70864742
Interaction	4.5206E+15	1	4.5206E+15	5.06344782	0.08762473	7.70864742
Within	3.5712E+15	4	8.9279E+14			
Total	1.7668E+16	7				

ANOVA (intact cells of treated and untreated plant leaves)

Source of Variation	*SS*	*df*	*MS*	*F*	*P-value*	*Fcrit*
Sample	8.5991E+13	1	8.5991E+13	1.81956529	0.2486702	7.70864742
Columns	4.0358E+13	1	4.0358E+13	0.85397307	0.40773177	7.70864742
Interaction	9.1962E+11	1	9.1962E+11	0.01945915	0.89579993	7.70864742
Within	1.8904E+14	4	4.7259E+13			
Total	3.163E+14	7				

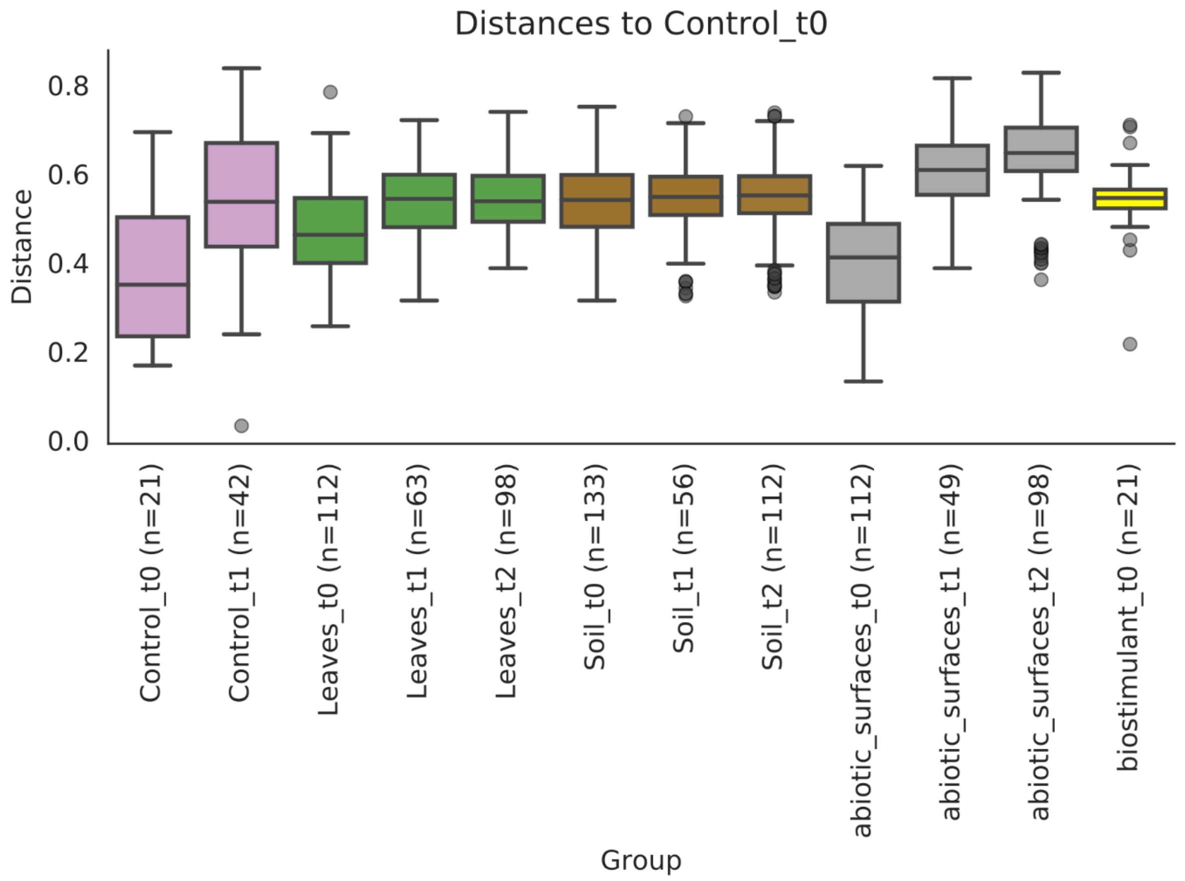

Supplementary Figure S4: Boxplot of weighted unifrac distances of diverse sample categories and sampling events (t0 = day 15, t1 = day 57, t2 = day 99).

Supplementary Table S4: Pairwise PERMANOVA results on weighted unifrac distances of diverse sample categories and sampling events (t0 = day 15, t1 = day 57, t2 = day 99).

PERMANOVA results (on weighted unifrac distances)	
method name	PERMANOVA
test statistic name	pseudo-F
sample size	135
number of groups	12
test statistic	11.5418
p-value	0.001
number of permutations	999

Group 1	Group 2	Sample size	Permutations	pseudo-F	p-value	q-value
Control_t0	Control_t1	13	999	4.049541	0.023	0.02372
Control_t0	Leaves_t0	23	999	7.79187	0.001	0.00154
Control_t0	Leaves_t1	16	999	10.83217	0.001	0.00154
Control_t0	Leaves_t2	21	999	18.18507	0.001	0.00154
Control_t0	Soil_t0	26	999	23.51612	0.001	0.00154
Control_t0	Soil_t1	15	999	15.69635	0.001	0.00154
Control_t0	Soil_t2	23	999	26.50874	0.001	0.00154
Control_t0	abiotic_surfaces_t0	23	999	3.522829	0.018	0.01886
Control_t0	abiotic_surfaces_t1	14	999	15.52368	0.003	0.00374
Control_t0	abiotic_surfaces_t2	21	999	40.92028	0.001	0.00154
Control_t0	biostimulant_t0	10	999	5.834078	0.009	0.00958

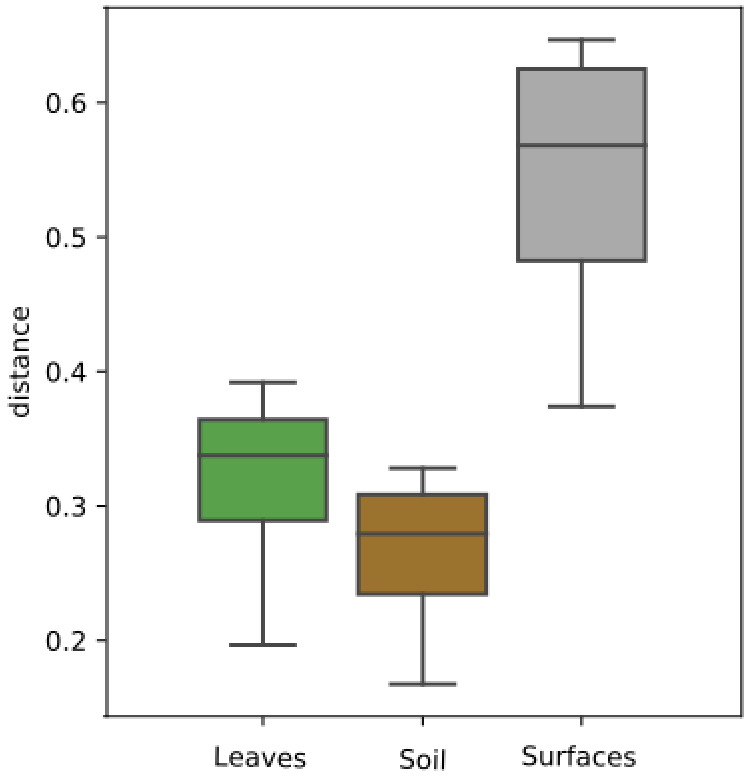

Supplementary Figure S5: Boxplot of pairwise weighted unifrac distance comparisons according to the main sampling environments plant leaves, soil and desiccator surfaces.

Supplementary Table S5: Statistics on multiple and pairwise group comparisons of weighted unifrac distances (Figure S5 and Figure S7).

Multiple group tests		
	H	P value
Kruskal Wallis test	0.6	0.438578

Pairwise group comparison tests				
Group A	Group B	Mann-Whitney U	P-value	FDR P-value
Untreated	biostimulant	3	0.698535	0.698535

Pairwise group comparison tests				
Group A	Group B	Mann-Whitney U	P-value	FDR P-value
Leaves	Surfaces	1	0.060602	0.082639
Soil	Leaves	4	0.312321	0.360371
Soil	Surfaces	0	0.030383	0.045574
Soil	biostimulant	3	0.723674	0.723674

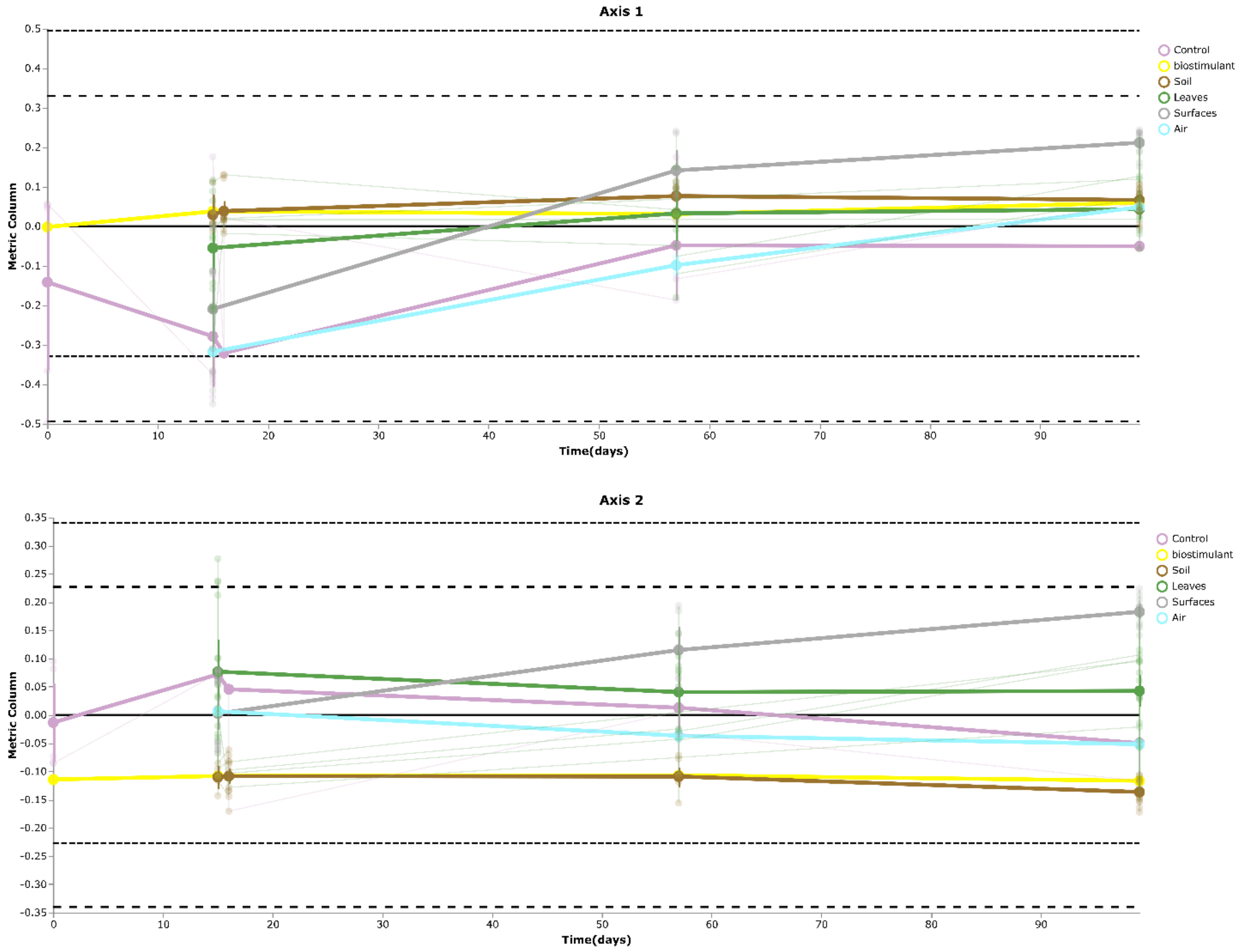

Supplementary Figure S6: Volatility plot of weighted unifrac distances for the sampling categories controls, biostimulant, soil, plant leaves, desiccator surfaces and air along PCoA Axis 1 (upper panel) and PCoA Axis 2 (lower panel).

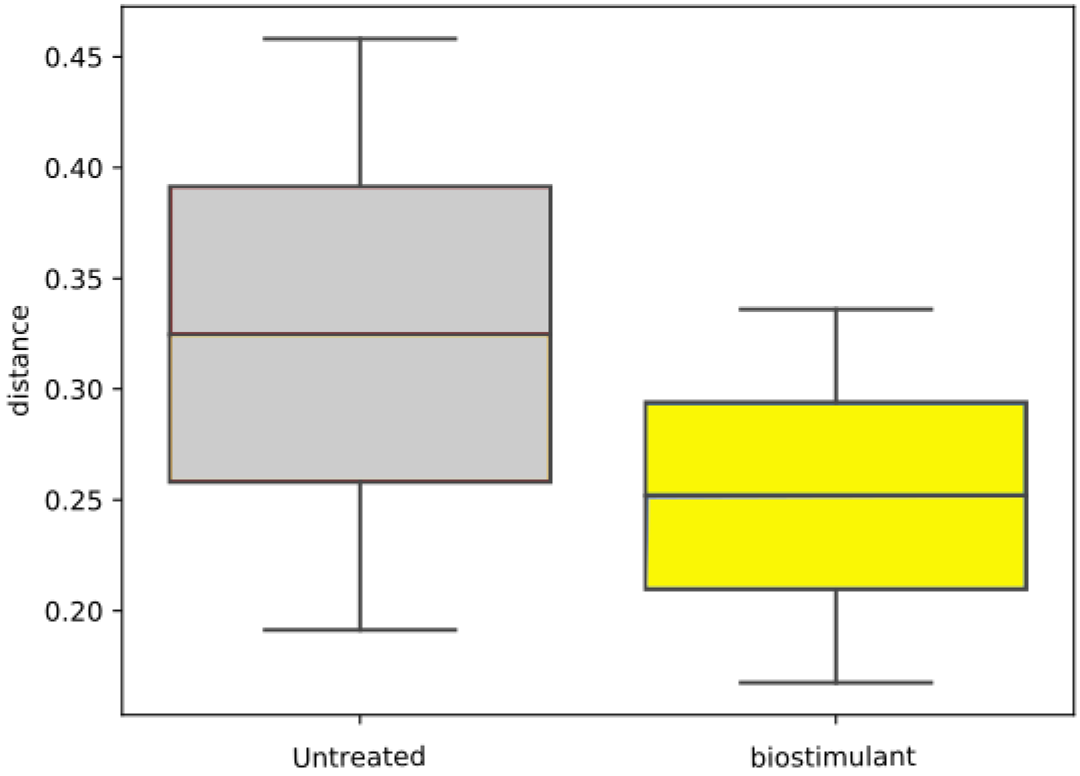

Supplementary Figure S7: Boxplot of pairwise weighted unifrac distance comparisons according to a treatment with the biostimulant.

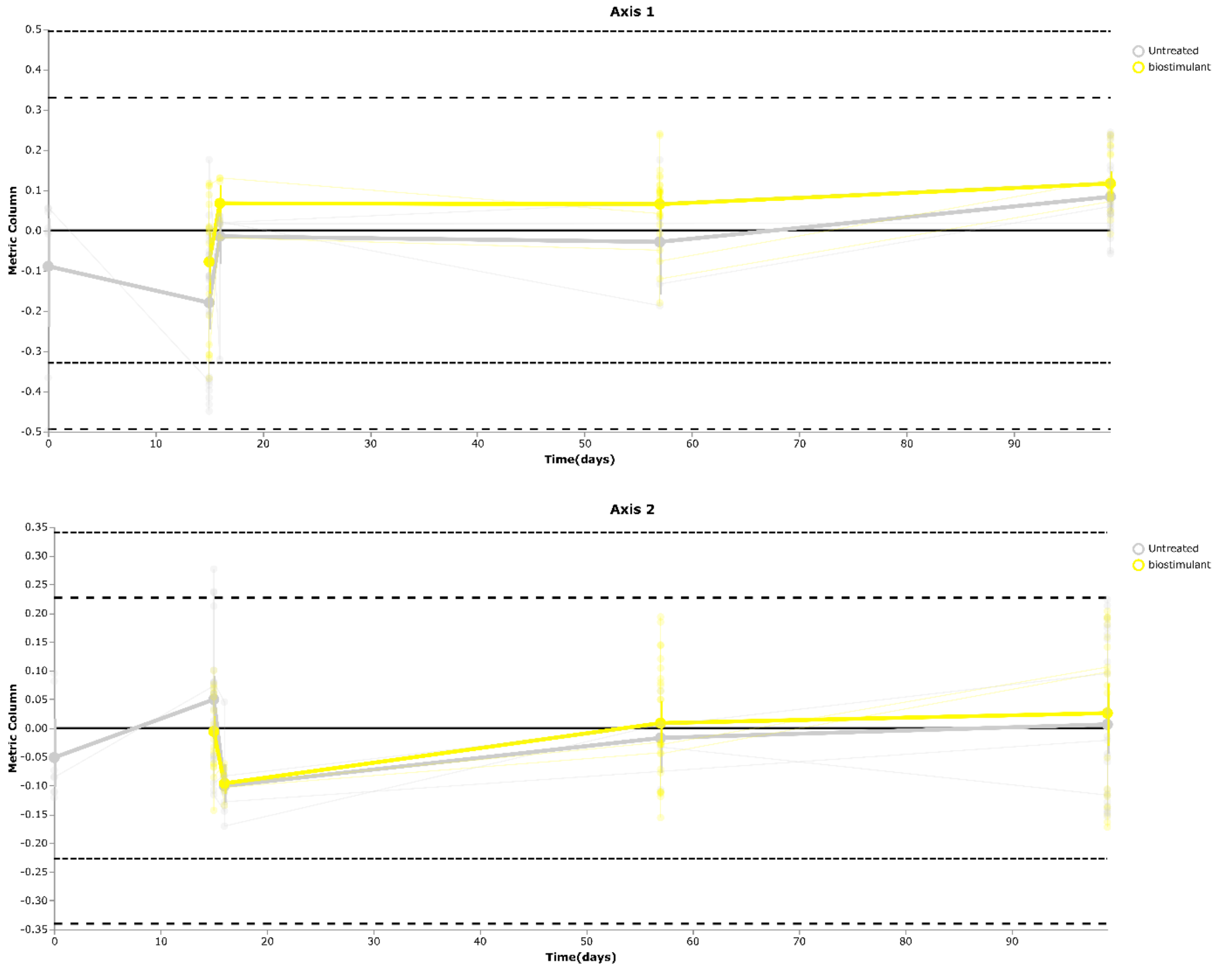

Supplementary Figure S8: Volatility plot of weighted unifrac distances for samples treated with the biostimulant and untreated (sterile and tap water) samples along PCoA Axis 1 (upper panel) and PCoA Axis 2 (lower panel).

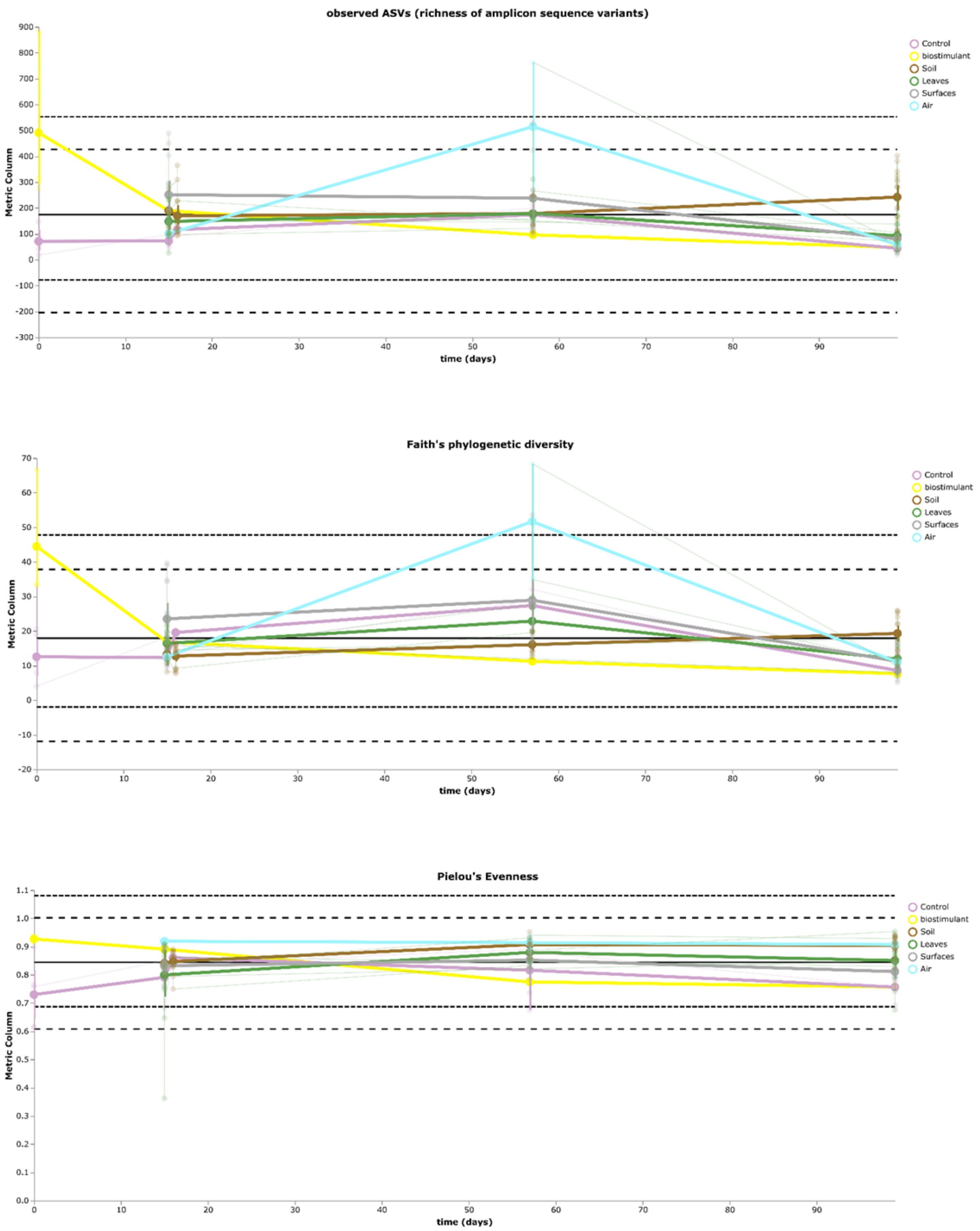

Supplementary Figure S9: Volatility plots of selected alpha diversity metrics (Shannon diversity, ASV richness, Faith's phylogenetic diversity, and Pielou's evenness).

Supplementary Table S6: Wilcoxon signed-rank test of pairwise differences between biostimulant treated and untreated samples. Pairwise difference test on shannon diversity of treated and untreated samples

	W (Wilcoxon signed-rank test)	P-value	FDR P-value
Group			
biostimulant	3	0.465209	0.465209
Untreated	0	0.043114	0.086229

Supplementary Table S7: Two-way ANOVA on the proportion of biostimulant typical ASVs (amplicon sequence variants) on desiccator surfaces and plant leaves.

ANOVA (proportion on desiccator surfaces over time)

Source of Variation	*SS*	*df*	*MS*	*F*	*P-value*	*Fcrit*
Sample	0.027641883	1	0.027641883	2.772923299	0.107022717	4.195971819
Columns	0.496630695	1	0.496630695	49.82000812	1.11803E-07	4.195971819
Interaction	0.03016582	1	0.03016582	3.02611463	0.092917653	4.195971819
Within	0.279117969	28	0.009968499			
Total	0.833556367	31				

ANOVA (proportion on plant leaves over time)

Source of Variation	*SS*	*df*	*MS*	*F*	*P-value*	*Fcrit*
Sample	0.005460125	1	0.005460125	0.111153179	0.741318881	4.195971819
Columns	0.138338	1	0.138338	2.816182494	0.104447034	4.195971819
Interaction	0.0389205	1	0.0389205	0.792314699	0.380985695	4.195971819
Within	1.37543075	28	0.049122527			
Total	1.558149375	31				

Supplementary Table S8: Mann-Whitney-Wilcoxon test on the proportion of potential pathogens according to BugBase.

Mann-Whitney-Wilcoxon Test (proportion of potential pathogens)				
Source of Variation	biostimulant treatment	untreated	P-value	FDR-corrected p-value
number of samples	78	80	0.0063661	0.0063661
relative abundance with trait (mean)	0.2251937	0.3131804		
relative abundance with trait (median)	0.1368009	0.2779227		
standard deviation	0.2195944	0.2283417		

Supplementary Table S9: Spearman's correlation of potential pathogens with time according to BugBase.

Spearman's Correlation for potential pathogens with time		
Treatment type	Correlation Estimate	P-value
biostimulant	-0.06176276	0.591143
untreated	0.3242281	0.003345765

Supplementary Table S10: List of potential pathogens according to BugBase.

Acidovorax caeni	*Cupriavidus*	*Lysobacter*	*Pseudomonas veronii*
Acidovorax facilis	*Delftia*	*Mannheimia*	*Pseudomonas viridiflava*
Acinetobacter	*Dickeya*	*Marinimicrobium*	*Ralstonia*
Acinetobacter guillouiae	*Dokdonella*	*Methylibium*	*Ramlibacter*
Acinetobacter johnsonii	*Dyella*	*Methylobacillus*	*Rheinheimera*
Acinetobacter lwoffii	*Enhydrobacter*	*Methylocaldum*	*Rhodanobacter*
Acinetobacter rhizosphaerae	*Enhydrobacter aerosaccus*	*Microbulbifer*	*Salinispora*
Acinetobacter schindleri	*Enterobacter*	*Microbulbifer elongatus*	*Salinispora tropica*
Acinetobacter venetianus	*Erwinia*	*Microvirgula aerodenitrificans*	*Salmonella*
Actinobacillus	*Erwinia dispersa*	*Moraxella*	*Salmonella enterica*
Actinobacillus parahaemolyticus	*Erwinia soli*	*Oxalobacter*	*Serratia*
Aggregatibacter	*Erwinia toletana*	*Perlucidibaca*	*Serratia marcescens*
Aggregatibacter segnis	*Haemophilus*	*Photobacterium*	*Shewanella*
Alcanivorax	*Haemophilus parainfluenzae*	*Photobacterium rosenbergii*	*Stenotrophomonas*
Alkanindiges	*Halomonas*	*Plesiomonas*	*Stenotrophomonas acidaminiphila*
Arenimonas	*Halorhodospira*	*Polynucleobacter*	*Stenotrophomonas geniculata*
Brenneria quercina	*Herminiimonas*	*Providencia*	*Stenotrophomonas retroflexus*
Burkholderia	*Hylemonella*	*Pseudidiomarina*	*Thermomonas*
Burkholderia andropogonis	*Idiomarina*	*Pseudomonas*	*Thermomonas fusca*
Burkholderia bryophila	*Janthinobacterium*	*Pseudomonas alcaligenes*	*Trabulsiella*
Burkholderia tuberum	*Janthinobacterium lividum*	*Pseudomonas balearica*	*Trabulsiella farmeri*
Cellvibrio	*Klebsiella*	*Pseudomonas citronellolis*	*Variovorax*
Citrobacter	*Leptothrix*	*Pseudomonas fragi*	*Variovorax paradoxus*
Collimonas	*Luteibacter rhizovicinus*	*Pseudomonas nitroreducens*	*Verminephrobacter*
Comamonas	*Luteimonas*	*Pseudomonas stutzeri*	*Vibrio rumoiensis*
Comamonas terrigena	*Luteimonas mephitis*	*Pseudomonas umsongensis*	*Yersinia*

Supplementary Table S11: PICRUSt predicted KEGG functions > 0.1% for the biostimulant.

KEGG functions related to detected metabolites are indicated in grey color.

KEGG pathway	proportion >0.1 % in biostimulant
Transporters	6.27
ABC transporters	3.82
General function prediction only	3.58
DNA repair and recombination proteins	2.31
Purine metabolism	1.88
Two-component system	1.80
Ribosome	1.76
Peptidases	1.58
Oxidative phosphorylation	1.44
Function unknown	1.44
Secretion system	1.42
Transcription factors	1.34
Bacterial motility proteins	1.31
Arginine and proline metabolism	1.30
Pyrimidine metabolism	1.29
Amino acid related enzymes	1.24
Pyruvate metabolism	1.14
Glycolysis / Gluconeogenesis	1.14
Methane metabolism	1.11
Chromosome	1.09
Carbon fixation pathways in prokaryotes	1.07
Amino sugar and nucleotide sugar metabolism	1.07
Others	1.03
Butanoate metabolism	1.03
Aminoacyl-tRNA biosynthesis	1.00
Ribosome Biogenesis	1.00
Porphyrin and chlorophyll metabolism	0.99
Propanoate metabolism	0.97
Glycine, serine and threonine metabolism	0.95
Other ion-coupled transporters	0.95
Transcription machinery	0.92
Valine, leucine and isoleucine degradation	0.91
Alanine, aspartate and glutamate metabolism	0.90
Lipid biosynthesis proteins	0.89
Citrate cycle (TCA cycle)	0.84
Chaperones and folding catalysts	0.83
Cysteine and methionine metabolism	0.81

Supplementary Table S11 cont.

KEGG pathway	proportion >0.1 % in biostimulant
Energy metabolism	0.81
Fatty acid metabolism	0.79
Valine, leucine and isoleucine biosynthesis	0.79
Protein folding and associated processing	0.77
DNA replication proteins	0.77
Glyoxylate and dicarboxylate metabolism	0.74
Pentose phosphate pathway	0.72
Translation proteins	0.71
Replication, recombination and repair proteins	0.71
Phenylalanine, tyrosine and tryptophan biosynthesis	0.70
Nitrogen metabolism	0.70
Homologous recombination	0.67
Tryptophan metabolism	0.66
Starch and sucrose metabolism	0.65
Lysine biosynthesis	0.63
Fatty acid biosynthesis	0.61
Bacterial secretion system	0.60
Mismatch repair	0.60
Histidine metabolism	0.59
Fructose and mannose metabolism	0.58
Pantothenate and CoA biosynthesis	0.58
Peptidoglycan biosynthesis	0.58
Benzoate degradation	0.56
Flagellar assembly	0.53
Terpenoid backbone biosynthesis	0.52
Lysine degradation	0.52
Protein export	0.52
Carbon fixation in photosynthetic organisms	0.50
beta-Alanine metabolism	0.50
Membrane and intracellular structural molecules	0.48
Glycerophospholipid metabolism	0.48
Aminobenzoate degradation	0.48
DNA replication	0.47
One carbon pool by folate	0.46
Pentose and glucuronate interconversions	0.46
Bacterial chemotaxis	0.46
Photosynthesis proteins	0.46

Supplementary Table S11 cont.

KEGG pathway	proportion >0.1 % in biostimulant
Cell cycle - Caulobacter	0.46
Base excision repair	0.46
Tyrosine metabolism	0.46
Folate biosynthesis	0.45
Signal transduction mechanisms	0.44
Limonene and pinene degradation	0.43
Galactose metabolism	0.43
RNA degradation	0.41
Translation factors	0.41
Protein kinases	0.40
Selenocompound metabolism	0.40
Photosynthesis	0.39
Phenylalanine metabolism	0.39
Nicotinate and nicotinamide metabolism	0.39
Geraniol degradation	0.39
Sulfur relay system	0.39
Glycerolipid metabolism	0.38
Thiamine metabolism	0.38
Ubiquinone and other terpenoid-quinone biosynthesis	0.38
Prenyltransferases	0.37
Glutathione metabolism	0.36
Glycosyltransferases	0.36
Inorganic ion transport and metabolism	0.35
C5-Branched dibasic acid metabolism	0.34
Streptomycin biosynthesis	0.33
Nucleotide excision repair	0.33
Pores ion channels	0.33
Sulfur metabolism	0.33
Lipopolysaccharide biosynthesis proteins	0.31
Biosynthesis of unsaturated fatty acids	0.31
Chloroalkane and chloroalkene degradation	0.30
Peroxisome	0.28
Caprolactam degradation	0.27
Naphthalene degradation	0.26
Amino acid metabolism	0.26
Lipopolysaccharide biosynthesis	0.24
Cyanoamino acid metabolism	0.24

Supplementary Table S11 cont.

KEGG pathway	proportion >0.1 % in biostimulant
Riboflavin metabolism	0.23
Cell motility and secretion	0.23
Drug metabolism - other enzymes	0.23
Inositol phosphate metabolism	0.22
PPAR signaling pathway	0.22
Cytoskeleton proteins	0.22
Other transporters	0.20
Toluene degradation	0.20
Vitamin B6 metabolism	0.19
Metabolism of cofactors and vitamins	0.18
Drug metabolism - cytochrome P450	0.18
Polycyclic aromatic hydrocarbon degradation	0.18
Tuberculosis	0.17
Ascorbate and aldarate metabolism	0.17
Metabolism of xenobiotics by cytochrome P450	0.17
Polyketide sugar unit biosynthesis	0.16
Carbohydrate metabolism	0.15
Sporulation	0.14
Synthesis and degradation of ketone bodies	0.14
Sphingolipid metabolism	0.14
Bisphenol degradation	0.14
Tetracycline biosynthesis	0.14
Biotin metabolism	0.13
Tropane, piperidine and pyridine alkaloid biosynthesis	0.13
Novobiocin biosynthesis	0.13
RNA polymerase	0.13
Nitrotoluene degradation	0.12
Plant-pathogen interaction	0.12
Taurine and hypotaurine metabolism	0.12
D-Glutamine and D-glutamate metabolism	0.11
Phenylpropanoid biosynthesis	0.11
Phosphatidylinositol signaling system	0.11
Restriction enzyme	0.11
Chlorocyclohexane and chlorobenzene degradation	0.11
Styrene degradation	0.11
Lipid metabolism	0.10
